# Mitochondrial DNA oxidative mutations are elevated in Mexican American women potentially implicating Alzheimer’s disease

**DOI:** 10.1038/s41514-022-00082-1

**Published:** 2022-04-04

**Authors:** Danielle Marie Reid, Robert C. Barber, Roland J. Thorpe, Jie Sun, Zhengyang Zhou, Nicole R. Phillips

**Affiliations:** 1 Microbiology, Immunology, and Genetics, School of Biomedical Sciences, UNT Health Science Center, Fort Worth, TX USA; 2 Pharmacology & Neuroscience, School of Biomedical Sciences, UNT Health Science Center, Fort Worth, TX USA; 3 Johns Hopkins Center for Health Disparities Solutions, Johns Hopkins Bloomberg School of Public Health, Baltimore, MD USA; 4 Biostatistics & Epidemiology, School of Public Health, UNT Health Science Center, Fort Worth, TX USA

**Keywords:** Genetics, Molecular biology

## Abstract

Mexican Americans (MAs) are the fastest-growing Hispanic population segment in the US; as this population increases in age, so will the societal burden of age-related diseases such as Alzheimer’s disease (AD). Mitochondrial DNA (mtDNA) damage may be implicated in MA AD risk since metabolic comorbidities are more prevalent in this group. Oxidative damage to guanosine (8oxoG) is one of the most prevalent DNA lesions and a putative indicator of mitochondrial dysfunction. Testing blood samples from participants of the Texas Alzheimer’s Research and Care Consortium, we found mtDNA 8oxoG mutational load to be significantly higher in MAs compared to non-Hispanic whites and that MA females are differentially affected. Furthermore, we identified specific mtDNA haplotypes that confer increased risk for oxidative damage and suggestive evidence that cognitive function may be related to 8oxoG burden. Our understanding of these phenomena will elucidate population- and sex-specific mechanisms of AD pathogenesis, informing the development of more precise interventions and therapeutic approaches for MAs with AD in the future.

## Introduction

The US aging population, characterized as individuals 65 years of age and older, is expanding at a rapid rate and is expected to grow for the next several decades reaching 88 million by 2050^1^. Correspondingly, the prevalence and number of age-related diseases, including Alzheimer’s disease (AD), are anticipated to increase and further burden our healthcare system^1^. AD is the sixth leading cause of death in the US, and while the prevalence of other leading causes of death in the US has decreased or remained about the same, the number of deaths due to AD has significantly increased from the year 2000 to 2019^1^.

AD is a fatal neurodegenerative disease attributed to neuronal damage and accumulating amyloid-β (Aβ) plaques in the brain, first implicating thinking, learning, and cognitive function^1,2^. The late-onset class of AD is sporadic, multifactorial, genetically complex (i.e., AD heritability has been estimated between 60% and 80% and is highly polygenic)^3^, and represents the majority (~90%) of total AD cases^4^. Moreover, it has been established that the progression of AD operates on a continuum from asymptomatic to AD-related dementia, with no distinct event denoting its onset; this progression is reflective of underlying accumulations of systemic and brain-specific pathology^1,5,6^. Early in progression there are two stages known as preclinical AD and mild cognitive impairment (MCI) due to AD that identifies individuals with AD brain changes without and with associated symptoms, respectively^1^.

Two of the most prominent pathological changes observed in AD are the accumulation of extracellular Aβ peptides producing senile plaques that block cell-cell signaling at synapses and the accumulation of intracellular hyperphosphorylated tau protein resulting in neurofibrillary tangles that inhibit the transportation of essential molecules^1–4,7^. However, the molecular and etiological events initiating AD pathologies remain to be determined^7^. Additional pathological changes exhibited in AD are mitochondrial dysfunction, chronic inflammation, and excess oxidative stress (OS)^4,7–9^. Mitochondrial stress, OS, and mitochondrial dysfunction are theorized to enhance AD pathology and play important roles in its pathogenesis^4,10^ and impaired mitochondrial function has been implicated in both AD and metabolic disease^2,4,7,8,11^.

Type-2 diabetes (T2D) has been shown to share similar pathological features with AD such as impaired glucose utilization, reduced mitochondrial activity, and both metabolic and mitochondrial dysfunction^7,11^. T2D is characterized by hyperglycemia caused by insulin resistance leading to insulin deficiency^7^. Pathophysiological features of T2D include islets of Langerhans cells presenting β cell loss and/or dysfunction, and spontaneous islet amyloid polypeptide aggregation^7^. Furthermore, there are reports that insulin regulates Aβ and tau protein metabolism, and there are numerous reviews discussing the established connections between insulin resistance, diabetes, and AD^7,12–14^.

Besides the existing general AD healthcare problems^1^, there are gaps in the scientific literature characterizing race/ethnicity-specific risk for disease development and progression of AD^15^. Recently, it has been recognized that ethnic/racial factors significantly impact biological and medical risk factors for AD^1,15,16^. In the US, there are more non-Hispanic whites (NHWs) living with AD than other racial/ethnic groups, although per-capita Hispanics are more likely to have AD^1,17^. Hispanic is a broad term, as this population encompasses a variety of ethnic subgroups that exhibit geographical and cultural differences^17^. The Hispanic population is represented by varying proportions of European, African, and Native American ancestry, and previous studies indicated the overall increased risk in Hispanics may be driven by a specific ethnic subgroup^17^. The presence of comorbid conditions (e.g., cardiovascular disease and diabetes) may explain in part, some of the disparity in AD prevalence^1^. In the US, Mexican Americans (MAs) represent majority of the Hispanic population, has one of the fastest-growing aging groups, and it is projected that by 2050 the number of aging MAs will triple, while rates of AD will grow six-fold among Hispanics^16,18,19^.

AD pathophysiology in the MA population seems to be distinct from NHWs. For example, the apolipoprotein E (APOE) allele ε4, which confers the largest risk for AD in NHWs, is far less significant in MAs. This may be in part due to the decreased frequency of the ε4 allele, combined with a smaller effect size^1,18,19^. Correspondingly, a recent study determined that APOE ε4 allele carrier status did not confer risk for MCI in MAs^20^. MAs clearly suffer from significant AD health disparities when compared to NHWs, including (1) earlier onset (~10 years) of cognitive impairment, (2) higher rates of missed diagnosis, (3) later diagnosis, and (4) increased prevalence of modifiable risk factors^1,16,18,19^. Depression, stroke, T2D, and obesity in the MA population are common risk factors for developing cognitive impairment that is more common in MAs, although the etiology remains unclear^16,21^. Lifestyle and/or metabolic health may contribute directly to age-related neurodegeneration^1^. Combined, these data emphasize the importance of conducting further studies to improve the diagnosis, treatment, and prevention of AD in the MA population.

Recently, there is growing evidence suggesting a correlation between common pathological changes in AD and oxidative damage to nucleic acids^4,8,22^. Mitochondria are highly vulnerable to oxidative DNA damage because they are predominant generators of reactive oxygen species (ROS), and their mitochondrial genome lacks histones and has a reduced capacity to repair DNA^7^. OS is a prominent contributor to Aβ aggregation and hyperphosphorylated tau, and numerous studies have provided evidence suggesting OS contributes to tau pathology because fatty acid oxidation accelerates tau polymerization^4,8,22^. In the central nervous system and peripheral tissues of AD patients, accumulation of ROS modifies the function and expression of antioxidant enzymes^4,8^. Also, high levels of DNA strand breaks were found in the hippocampus and cerebral cortex of AD brains^4^.

**Fig. 1:**
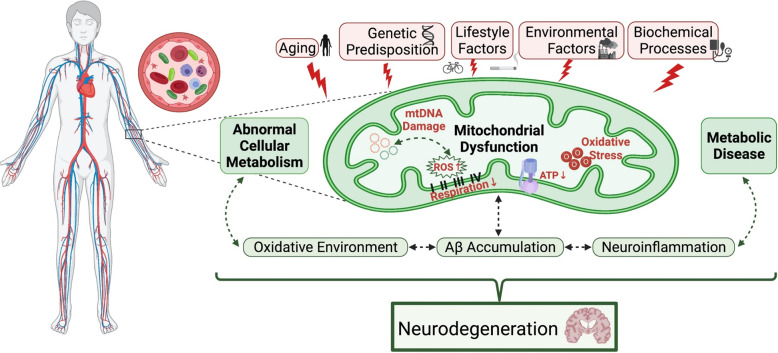
Graphical overview of global working hypothesis for risk factors and cellular/molecular processes that contribute to neurodegeneration. Modifiable and unmodifiable risk factors such as age, genetics, and lifestyle/environmental factors can induce elevated levels of ROS which could lead to mitochondrial and/or metabolic pathophysiology. This pathophysiology can contribute to and exacerbate an oxidative environment, neuroinflammation, and amyloid-beta accumulation that could ultimately promote neurodegeneration. This figure was created with BioRender.com.

Mitochondrial dysfunction causes an increased mtDNA somatic mutation rate, reduced energy metabolism, increased ROS, and intensifies the mitochondrial oxidative environment^2^. The most common forms of oxidative damage observed in AD brains are 8-oxo-2’-deoxyguanine (8oxodG) and 8-oxo-guanine (8oxoG)^4^. In the cortex and cerebellum of AD patients compared to controls, significantly higher levels of 8oxodG were observed in the ventricular CSF^23^. Elevated levels of both forms of oxidatively modified guanine have been demonstrated in the nDNA of AD brains when compared to age-matched controls^4^. Interestingly, Aβ is an important factor in mitochondrial dysfunction and increases ROS production in AD^4^. Mitochondrial dysfunction and excessive levels of Aβ can activate the mitochondrial permeability transition pore leading to the destruction of neurons with defective mitochondria^10^. Furthermore, it has been demonstrated in the hippocampal neurons of AD patients that Aβ decreases the activity of essential ETC enzymes and alters mitochondrial dynamics^4,8^. These enzymes are highly susceptible to oxidative damage and the reduced activity of key enzymes involved in intermediate metabolism is a characteristic of abnormal cerebral glucose utilization^7^. Mitochondrial-induced OS may play an important role in the progression and pathophysiological changes in the brain of AD because neurons and mitochondria are sensitive to OS-inducing mitochondrial dysfunction (Fig. 1).

Previously, our lab investigated the role of mitochondria in T2D and cognitive impairment in MAs through analyzing blood-based features of mitochondrial abnormalities (i.e., mtDNA copy number and cell-free mtDNA)^11^. The data suggested mitochondrial dysfunction assessed by mtDNA copy number was closely related to both T2D and cognitive impairment^11^. Here, our objective was to determine if abnormal mitochondrial function, indicated by oxidative DNA damage, differs between population (MA vs. NHW), as well as to evaluate the effects of sex, cognitive impairment, and T2D on AD risk. Using Illumina-based next-generation sequencing, we quantified oxidatively modified guanine residues in mtDNA. Our data show that 8oxoG mutational load is significantly higher in MAs than in NHWs and is associated with cognitive function, sex, and education. Particularly, the sex effect observed was moderated by population. Stratified analysis for 8oxoG mutational load in MAs suggests significant elevation when comparing MAs with AD to normal controls.

## Results

The descriptive statistics of the cohort are provided in Table 1. In both populations, MMSE, CDR sum, and years of education significantly differed between cognitive phenotypes as expected. Age was determined to significantly differ by cognitive diagnosis, and years of education was lower in the MA population. A Pearson correlation determined 8oxoG variant count did not significantly differ by age in the total cohort (Supplementary Fig. 3).

**Table 1:** Descriptive statistics of participants by population group and cognitive phenotype in the Texas Alzheimer’s Research and Care Consortium.

	NC	MCI	AD	*p* value^a^
*Total number of subjects*	328	127	104	
*Non-Hispanic whites*	153	43	64	
Age [CI]	70.39 ± 1.178	71.35 ± 1.421	71.7 ± 1.056	0.338
Sex (F) [*n*, %]	78, 51.0%	21, 48.8%	29, 45.3%	0.749
Mini Mental State Exam (MMSE) [CI]	29.11 ± 0.1759	27.63 ± 0.6223	21.53 ± 1.413	0.000^b^
Clinical Dementia Rating (CDR) Sum [CI]	0.007 ± 0.009032	1.163 ± 0.2181	5.344 ± 0.8515	0.000^c^
Years of education [CI]	16.07 ± 0.4063	14.56 ± 0.6597	15.11 ± 0.7524	0.001^d^
Diabetes (Y) [*n*, %]	59, 38.6%	18, 41.9%	22, 34.4%	0.726
Hyperlipidemia (Y) [*n*, %]	63, 41.2%	19, 44.2%	37, 57.8%	0.079
Obesity (Y) [*n*, %]	27, 17.6%	8, 18.6%	10, 15.6%	0.991
BMI, kg/m^2^ [CI]	27.331 ± 1.1778	27.272 ± 2.3284	27.394 ± 1.0624	0.996
*Mexican Americans*	175	84	40	
Age [CI]	67.62 ± 0.8156	69.88 ± 1.6912	73.38 ± 2.4848	0.000^e^
Sex (F) [*n*, %]	99, 56.6%	40, 47.6%	24, 60.0%	0.304
Mini Mental State Exam (MMSE) [CI]	28.14 ± 0.289	24.93 ± 0.787	19.88 ± 1.860	0.000^f^
Clinical Dementia Rating (CDR) Sum [CI]	0.006 ± 0.007897	1.113 ± 0.1560	5.738 ± 1.1827	0.000^g^
Years of education [CI]	11.05 ± 0.6598	8.77 ± 1.1486	9.75 ± 1.5467	0.002^h^
Diabetes (Y) [*n*, %]	79, 45.1%	32, 38.1%	19, 47.5%	0.487
Hyperlipidemia (Y) [*n*, %]	101, 57.7%	48, 57.1%	20, 50.0%	0.670
Obesity (Y) [*n*, %]	84, 48.0%	38, 45.2%	8, 20.0%	0.005^i^
BMI, kg/m^2^ [CI]	30.917 ± 0.9992	31.295 ± 1.5374	28.718 ± 1.6508	0.116

^a^The mean difference is significant at 0.05.

^b^NC vs. MCI 0.016, NC vs. AD 0.000, MCI vs. AD 0.000.

^c^NC vs. MCI 0.000, NC vs. AD 0.000, MCI vs. AD 0.000.

^d^NC vs. MCI 0.003, NC vs. AD 0.042, MCI vs. AD 0.542.

^e^NC vs. MCI 0.028, NC vs. AD 0.000, MCI vs. AD 0.017.

^f^NC vs. MCI 0.000, NC vs. AD 0.000, MCI vs. AD 0.000.

^g^NC vs. MCI 0.000, NC vs. AD 0.000, MCI vs. AD 0.000.

^h^NC vs. MCI 0.001, NC vs. AD 0.273, MCI vs. AD 0.540.

^i^NC vs. MCI 0.905, NC vs. AD 0.003, MCI vs. AD 0.021.

### Total 8oxoG variant count is significantly higher in MA females

**Fig. 2:**
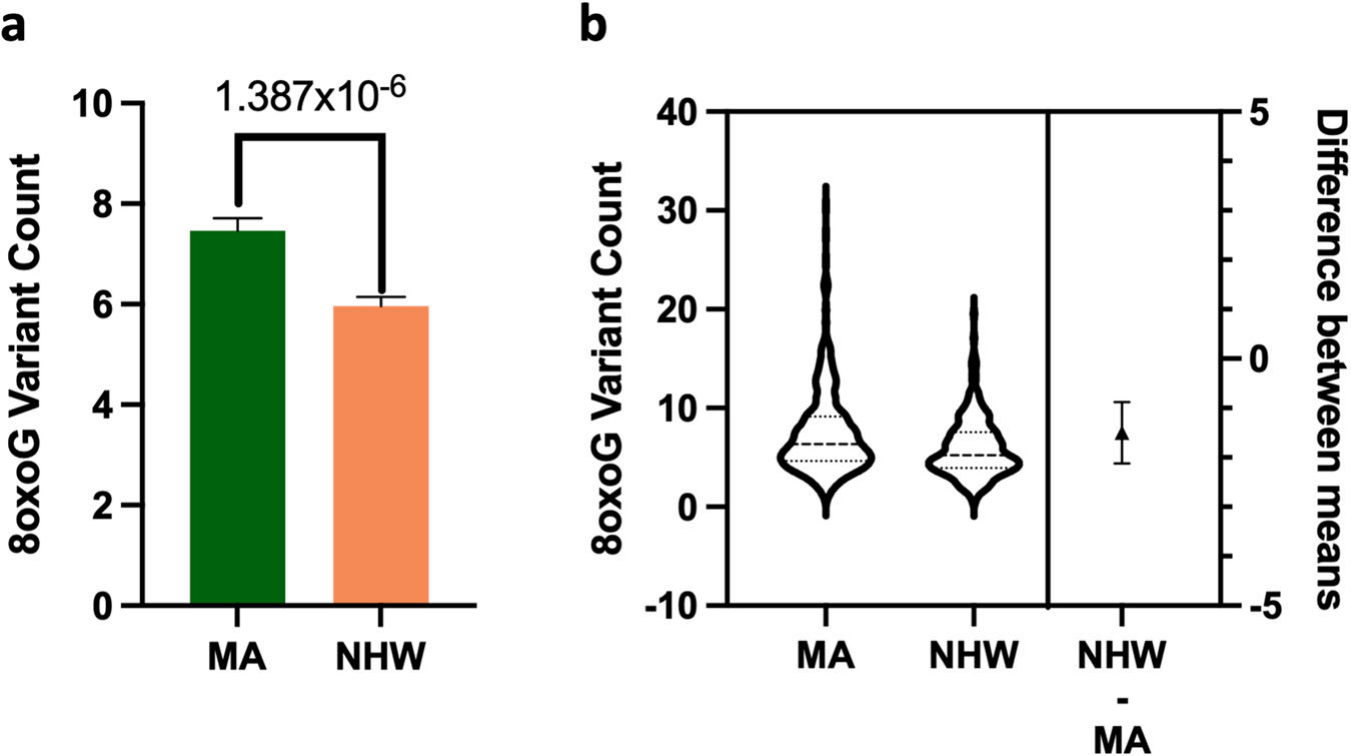
8oxoG variant count is significantly higher in Mexican American population. **a** Total 8oxoG variant count was assessed by population using a two-tailed Welch’s *t*-test (*n* = 559, *t*-statistic = 4.794, df = 558). Error bars represent standard error of the mean. **b** Violin plot showing the distribution of 8oxoG variant counts in Mexican American and non-Hispanic whites (*n* = 559) with effect size and confidence interval plotted on right *y*-axis. Dashed lines indicate the mean and dotted lines represent the 1st and 3rd quartile. The triangle represents the difference of the means, and the associated bar indicates the confidence interval.

**Fig. 3:**
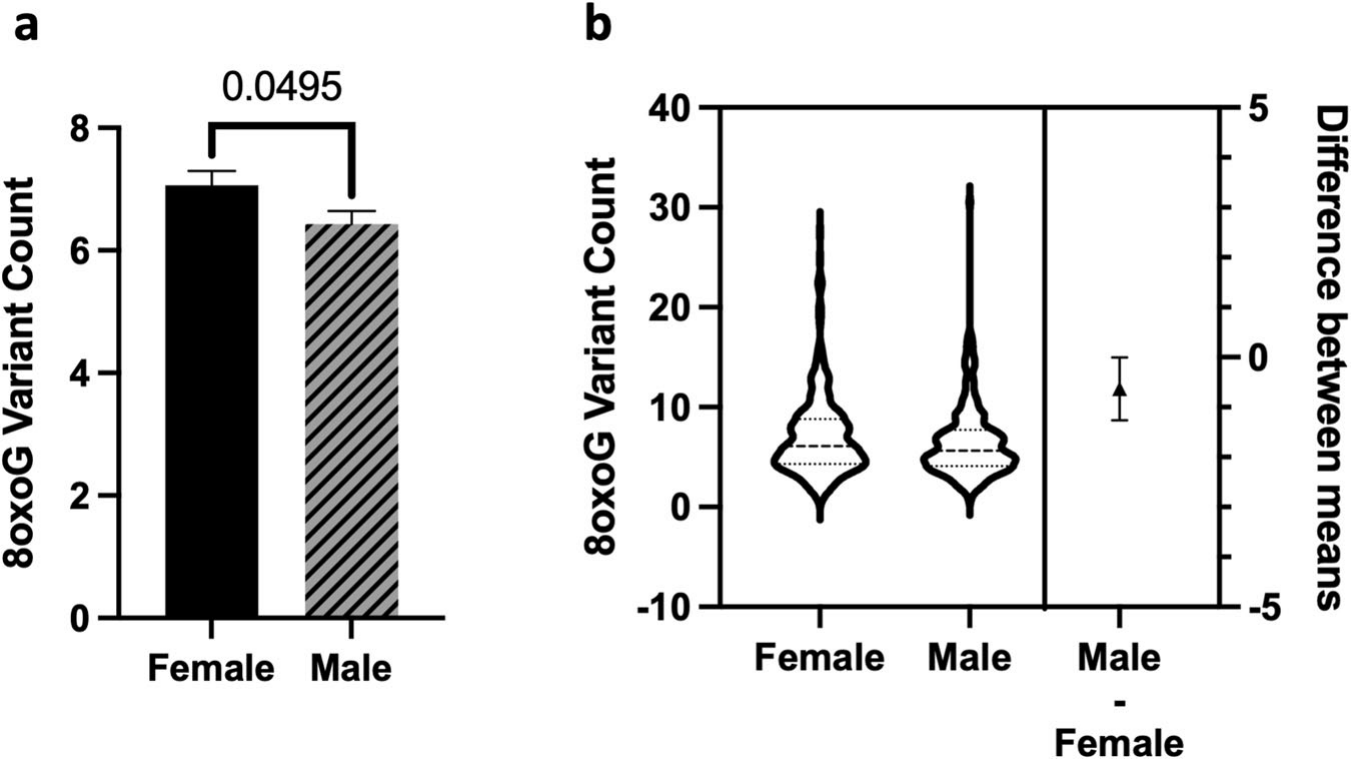
8oxoG variant count is significantly higher in females. **a** Sex differences in 8oxoG variant count were determined using a two-tailed Welch’s *t*-test (*n* = 559, *t*-statistic = 1.968, df = 558). Error bars represent standard error of the mean. **b** Violin plot showing the distribution of 8oxoG variant counts in females and males (*n* = 559) with effect size and confidence interval plotted on right *y*-axis. Dashed lines indicate the mean and dotted lines represent the 1st and 3rd quartile. The triangle represents the difference of the means, and the associated bar indicates the confidence interval.

**Fig. 4:**
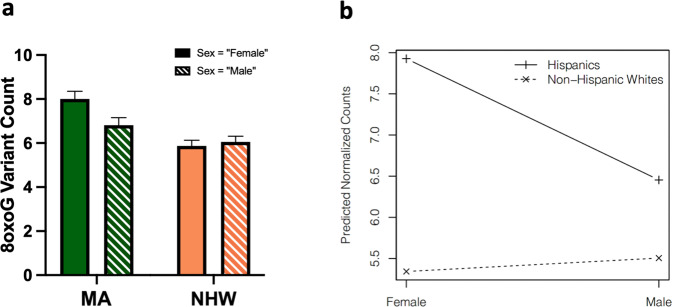
Population-by-sex interaction associated with total 8oxoG variant count shows MA females have elevated 8oxoG counts. **a** Bar graph representing total 8oxoG variant count by population and sex as tested using a two-way ANOVA (*n* = 559, *p* = 0.0297, F-statistic = 4.75, df = 557) to determine if a population × sex interaction existed. **b** Interaction plot of predicted 8oxoG variant counts by sex in NHWs and MAs. Error bars represent standard error of the mean.

Total 8oxoG variant count was significantly higher in the MA population compared to NHWs; mean = 7.46 and 5.96, respectively (Fig. 2). In addition, female subjects had a higher 8oxoG variant count than males; mean = 7.06 and 6.43, respectively (Fig. 3). The more comprehensive multiple linear regression model (Table 2) pointed to a significant interaction effect between population and sex related to 8oxoG variant count; *p* = 0.01458, MA females being higher; *p* = 0.0297 (Fig. 4); in addition, years of education was identified as a significant factor (positive association). No other variables included in the multiple linear regression model demonstrated associated statistical significance (BMI, APOE, diabetes, cognition, age, population × education).

**Table 2:** 8oxoG variant count and cognitive status (NC vs. MCI or AD) multiple linear regression model prediction considering population interaction effect with both sex and education.

Variable	Coefficient	SE	*t*-statistic	*p* value^a^
Constant	2.89096	2.21327	1.306	0.19204
Population (with respect to NHW)	–0.52522	1.46648	–0.358	0.72037
Cognitive status (with respect to AD)	0.56007	0.45113	1.241	0.21497
Cognitive status (with respect to MCI)	0.4515	0.40816	1.106	0.26914
Sex (with respect to male)	–1.42142	0.44486	–3.195	***0.00148***
Diabetes (with respect to “Yes”)	–0.35714	0.33836	–1.056	0.29166
Years of education	0.14335	0.04556	3.146	***0.00174***
APOE ε2/ε2	–2.3329	2.6982	–0.865	0.38763
APOE ε2/ε3	0.64292	0.81025	0.793	0.42785
APOE ε2/ε4	0.47953	2.23482	0.215	0.83018
APOE ε3/ε3	0.61844	0.61248	1.01	0.31308
APOE ε3/ε4	0.36641	0.66434	0.552	0.58149
APOE ε4/ε4	0.65167	0.91108	0.715	0.47475
BMI	0.03357	0.02468	1.36	0.17424
Age	0.0308	0.0252	1.222	0.22207
Interaction: NHW × Male “Yes”	1.58842	0.64817	2.451	***0.01458***
Interaction: NHW × Years of education	–0.15153	0.09808	–1.545	0.12291
*R*-squared	0.08275		*p* value	5.87e–05
Adjusted *R*-squared	0.05567		df	16 and 542
*F*-statistic	3.056		Sample *n*	559

^a^Italics and bolding indicate a *p* value of significance, while italics alone indicate a *p* value approaching significance (alpha = 0.05).

In a subsequent multiple linear regression analysis, we investigated the potential interaction between diabetes and cognitive status, in which we did not observe significant effects (Supplementary Table 1). We also derived the count of variants for each individual that corresponded to 8oxoG “hotspots” (i.e., frequently observed variants at certain locations within the mitochondrial genome) shown in Supplementary Fig. 4. In these “hotspot” analyses, we did not observe the same trends, and thus the metric proved to be generally less informative (Supplementary Figs. 4–5 and Supplementary Tables 6–13). In addition, in the NHW population we observed associations between 8oxoG “hotspot” variant count and APOE status (Supplementary Tables 12–13), which was not observed in the MA population (Supplementary Tables 10 and 11).

### Population-specific effects on 8oxoG variant count

**Fig. 5:**
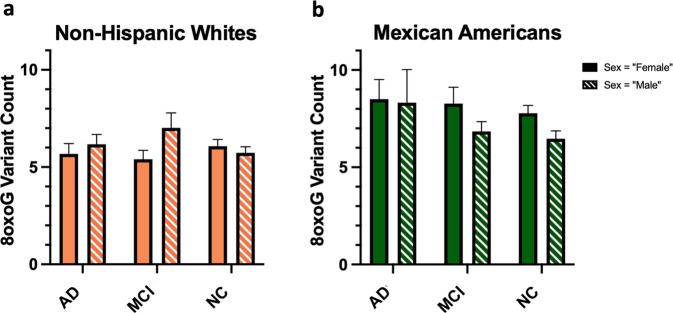
8oxoG variant count by cognitive phenotype in each population. **a** Bar graph of 8oxoG count by cognition in NHWs tested using a two-way ANOVA (*n* = 260). **b** Bar graph of 8oxoG count by cognition in MAs tested using a two-way ANOVA (*n* = 299) to determine if a cognition × sex interaction existed in each population. Error bars represent standard error of the mean.

As expected, based on the previous multiple linear regression analyses, 8oxoG variant count was significantly associated with sex (females higher) for MAs as shown in Table 3. However, interestingly, cognitive status of AD was in marginally significant association with 8oxoG variant count (shaded row, Table 3; bar graph provided in Fig. 5), but this trend was not observed in NHWs (shaded row, Table 4). Two-way ANOVAs in NHWs did not show significance; however, in MAs there was significance for sex *F*(1,295) = 5.8 and *p* = 0.0166 (Fig. 5). No other variables were associated with 8oxoG variant count in the MA population. BMI and age were marginally significant (both positive) in association with 8oxoG variant count in NHWs (Table 4); no other variables were associated with 8oxoG variant count. Another intriguing result is the significant positive association of education with 8oxoG variant count that is limited to the MA population (Table 3).

**Table 3:** Multiple linear regression results for 8oxoG variant count within Mexican Americans.

Variable	Coefficient	SE	*t*-statistic	*p* value^a^
Constant	5.12074	3.63974	1.407	0.16054
Cognitive status (with respect to AD)^b^	1.4363	0.80455	1.785	*0.07528*
Cognitive status (with respect to MCI)	0.75592	0.59613	1.268	0.20581
Sex (with respect to male)	–1.39809	0.52709	–2.652	***0.00844***
Diabetes (with respect to “Yes”)	–0.27268	0.52981	–0.515	0.60719
APOE ε2/ε2	–1.69728	4.72412	–0.359	0.71965
APOE ε2/ε3	0.2323	2.17461	0.107	0.91501
APOE ε3/ε3	–0.46508	1.9656	–0.237	0.81313
APOE ε3/ε4	–0.72004	2.00661	–0.359	0.71998
APOE ε4/ε4	–0.82949	2.92419	–0.284	0.77687
BMI	0.02671	0.03926	0.68	0.49687
Years of education	0.14462	0.05415	2.671	***0.008***
Age	0.01253	0.03924	0.319	0.74983
*R*-squared	0.05857		*p* value	0.1297
Adjusted *R*-squared	0.01907		df	12 and 286
*F*-statistic	1.483		Sample *n*	299

^a^Italics and bolding indicate a *p* value of significance, while italics alone indicate a *p* value approaching significance (alpha = 0.05).

^b^Shaded for emphasis when comparing to the table for NHW regression results (Table 4).

**Table 4:** Multiple linear regression results for 8xoG variant count within non-Hispanic whites.

Variable	Coefficient	SE	*t*-statistic	*p* value^a^
Constant	0.59072	2.81035	0.21	0.8337
Cognitive status with respect to AD^b^	–0.23042	0.48975	–0.47	0.6384
Cognitive status (with respect to MCI)	0.03883	0.54101	0.072	0.9428
Sex (with respect to male)	0.18523	0.37372	0.496	0.6206
Diabetes (with respect to “Yes”)	–0.55416	0.40735	–1.36	0.1749
APOE ε2/ε2	–4.04738	2.99107	–1.353	0.1772
APOE ε2/ε3	0.30547	0.80613	0.379	0.7051
APOE ε2/ε4	0.29945	1.7921	0.167	0.8674
APOE ε3/ε3	0.89858	0.54737	1.642	0.1019
APOE ε3/ε4	0.76197	0.65579	1.162	0.2464
APOE ε4/ε4	1.28886	0.81301	1.585	0.1142
BMI	0.04855	0.02883	1.684	*0.0935*
Years of education	–0.02771	0.07105	–0.39	0.6969
Age	0.05584	0.03101	1.801	*0.073*
*R*-squared	0.04846		*p* value	0.488
Adjusted *R*-squared	–0.001829		df	13 and 246
*F*-statistic	0.9636		Sample *n*	260

^a^Italics and bolding indicate a *p* value of significance, while italics alone indicate a *p* value approaching significance (alpha = 0.05).

^b^Shaded for emphasis when comparing to the table for MA regression results (Table 3).

Additional multiple linear regression analyses using cognition as a binary predictive variable (where MCI and AD are combined into cognitive impairment, CI, and NC is normal controls) were conducted (Supplementary Tables 2–5, 7, 9, 11, and 13); the higher 3-category resolution shown in Table 3 (AD/MCI/NC) revealed a potential effect of AD on 8oxoG variant count in MAs, but the effect is not observable in the CI/NC regression analyses since it is diluted by the presence of MCI (Supplementary Table 4).

### Haplogroup-associated elevation and depression of 8oxoG variant count

**Fig. 6:**
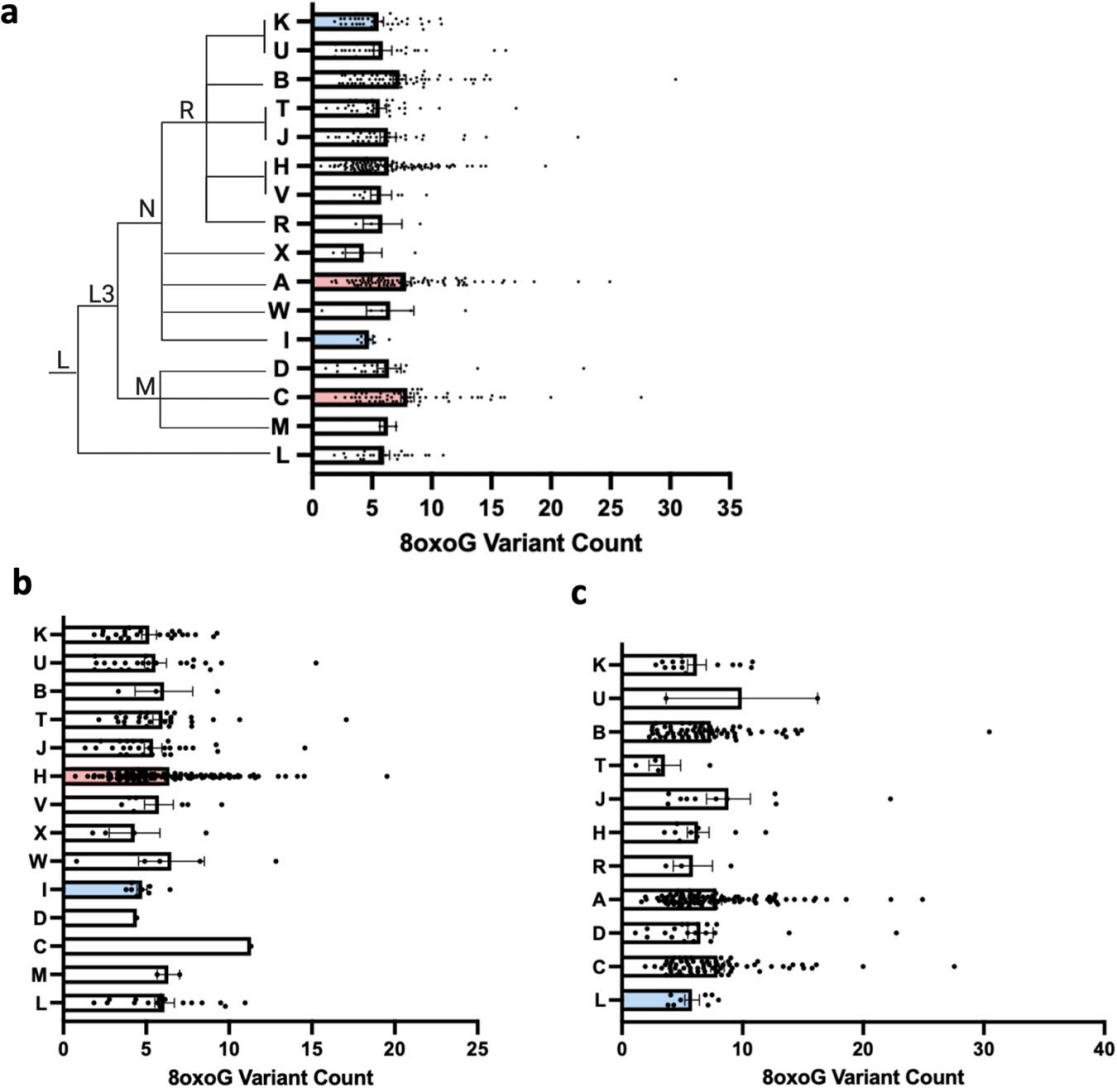
8oxoG variant count by mitochondrial haplogroup. **a** Differences in total 8oxoG variant count by mitochondrial haplogroup of the cohort was assessed using Welch’s *t*-test (*n* = 560). **b** Total 8oxoG variant count by mitochondrial haplogroup in NHW participants was assessed using Welch’s *t*-test (*n* = 261). **c** Differences in 8oxoG variant count between mitochondrial groups in the MA population was determined by performing Welch’s *t*-test (*n* = 299). Pink bars indicate significantly higher 8oxoG variant count and blue bars indicate significantly lower 8oxoG variant count. Error bars represent standard error of the mean. The mitochondrial haplogroup tree was illustrated based off the RSRS-oriented mtDNA tree build 17 from PhyloTree_mt_ to include only macrohaplogroups and sub-macrohaplogroups represented in our cohort^61^.

Based on the Welch two-sided *t*-test, we observed haplogroup effects on 8oxoG variant burden within the combined cohort. Haplogroups A and C exhibited elevated 8oxoG variant counts (Fig. 6a and Table 5). Conversely, haplogroups I and K exhibited lower 8oxoG variant counts (Fig. 6a and Table 5). For population stratified inference, in the NHW population, using Welch’s *t*-test, our results demonstrate haplogroup H displayed higher 8oxoG variant counts (Fig. 6b and Table 5). Haplogroup I among NHWs showed reduced 8oxoG variant counts (Fig. 6b and Table 5). In the MA population, Welch’s *t*-test reported haplogroup L had significantly reduced 8oxoG variant counts when compared to all other haplogroups observed in the MA population (Fig. 6c and Table 5).

**Table 5:** Mitochondrial DNA haplogroup-associated 8oxoG variant count mean within the combined cohort (NHW + MA; *n* = 560), MAs alone (*n* = 299), and NHWs alone (*n* = 261).

	Reference haplogroup mean	Non-reference haplogroup mean	*t*-statistic	df	95% CI [LL, UL]	*p* value^a^
*Combined reference haplogroup*						
Haplogroup A	7.838462	6.51901	2.8867	132.94	[0.41535, 2.2236]	0.004545
Haplogroup B	7.303423	6.680043	1.1141	79.498	[–0.49026, 1.7370]	0.2686
Haplogroup C	7.9767	6.591353	2.4044	77.106	[0.23809, 2.5326]	0.0186
Haplogroup D	6.41393	6.7679	–0.34926	21.068	[–2.4612, 1.7533]	0.7304
Haplogroup H	6.364743	6.862089	–1.461	238.38	[–1.1679, 0.17326]	0.1453
Haplogroup I	4.752968	6.783636	–5.9126	11.613	[–2.7818, –1.2796]	8.127E–05
Haplogroup J	6.338881	6.784038	–0.65586	40.693	[–1.8162, 0.92590]	0.5156
Haplogroup K	5.555933	6.841888	–2.9575	51.28	[–2.1588, –0.41315]	0.004681
Haplogroup L	6.015808	6.790599	–1.635	32.557	[–1.7394, 0.18980]	0.1117
Haplogroup M	6.339475	6.756114	–0.59872	1.1166	[–7.3496, 6.5163]	0.6479
Haplogroup R	6.759379	5.872131	0.54395	2.0397	[–6.0017, 7.7762]	0.6401
Haplogroup T	6.81632	5.664707	1.9933	34.475	[–0.021895, 2.3251]	0.05419
Haplogroup U	6.794277	5.906105	1.1589	26.368	[–0.68615, 2.4625]	0.2569
Haplogroup V	6.767161	5.76442	1.1311	6.4231	[–1.1322, 3.1377]	0.2985
Haplogroup W	6.756741	6.519856	0.11912	4.0531	[–5.2561, 5.7298]	0.9109
Haplogroup X	6.772306	4.297114	1.6096	3.0671	[–2.3588, 7.3092]	0.2039
*MA reference haplogroup*						
Haplogroup A	7.838462	7.270255	1.0848	202.54	[–0.46460, 1.6010]	0.2793
Haplogroup B	7.361145	7.487292	–0.20331	97.394	[–1.3575, 1.1052]	0.8393
Haplogroup C	7.925182	7.331154	0.95278	98.058	[–0.64322, 1.8313]	0.343
Haplogroup D	6.513252	7.528179	–0.94198	21.341	[–3.2534, 1.2236]	0.3567
Haplogroup H	6.320519	7.495663	–1.2566	9.3373	[–3.2791, 0.92881]	0.2394
Haplogroup J	8.822136	7.413168	0.7684	9.3447	[–2.7158, 5.5338]	0.4612
Haplogroup K	6.207265	7.521843	–1.6302	16.132	[–3.0229, 0.39373]	0.1224
Haplogroup L	5.81628	7.505487	–2.5543	9.6501	[–3.1700, –0.20844]	0.0294
Haplogroup R	7.476387	5.872131	0.97675	2.0968	[–5.1588, 8.3674]	0.4276
Haplogroup T	7.513174	3.56018	2.9696	3.2244	[–0.12126, 8.0272]	0.05397
Haplogroup U	7.443624	9.935283	–0.39662	1.0031	[–81.727, 76.743]	0.7595
*NHW reference haplogroup*						
Haplogroup B	6.072026	5.944759	0.072626	2.0441	[–7.2587, 7.5133]	0.9486
Haplogroup H	6.368296	5.628957	1.9889	218.16	[0.0067015, 1.4720]	0.04796
Haplogroup I	4.752968	5.983953	–3.465	13.256	[–1.9970, –0.46450]	0.004076
Haplogroup J	5.419158	6.007037	–1.0462	33.322	[–1.7307, 0.55496]	0.303
Haplogroup K	5.17599	6.02422	–1.7433	32.388	[–1.8388, 0.14238]	0.09076
Haplogroup L	6.104487	5.934498	0.27424	20.689	[–1.1202, 1.4602]	0.7866
Haplogroup M	6.339475	5.943185	0.56521	1.1507	[–6.1779, 6.9705]	0.6622
Haplogroup T	5.941546	5.98848	–0.075832	30.492	[–1.3101, 1.2162]	0.94
Haplogroup U	5.983957	5.555742	0.62167	25.685	[–0.98851, 1.8449]	0.5396
Haplogroup V	5.951232	5.76442	0.20969	6.5517	[–1.9494, 2.3230]	0.8403
Haplogroup W	5.935018	6.519856	–0.29383	4.0673	[–6.0752 4.9055]	0.7833
Haplogroup X	5.971888	4.297114	1.0874	3.0859	[–3.1504, 6.5000]	0.3544

^a^Nominal *p* values.

## Discussion

AD was discovered over a century ago, and through research, our understanding of the disease has exponentially grown. However, there are many gaps in our knowledge, particularly with respect to how this disease affects individuals from different ethnic/racial backgrounds. Our group investigated peripheral levels of mitochondrial 8oxoG, a characteristic of mitochondrial dysfunction, and its association with cognitive impairment, T2D, and comorbidity (cognitive impairment and T2D) within the MA population compared to NHWs. We hypothesized the MA population would demonstrate higher levels of mitochondrial oxidative damage due to the number of comorbid conditions burdening this population, such as cardiovascular disease, diabetes, and depression^1^. Overall, our results demonstrate that 8oxoG variant count was significantly higher in MAs compared to NHWs, and this effect was largely driven by MA females. In subsequent regression analyses, we observed that 8oxoG variant count is suggestively associated with AD cognitive status (compared to control), particularly in MAs. Intriguingly, this analysis also revealed a positive association of 8oxoG variant count with education, warranting further investigation of biological and/or environmental influencers of 8oxoG.

The level of 8oxoG variant count in the mitochondrial genome was significantly higher in MAs compared to NHWs, which may be because MAs are at increased risk for metabolic disorders. Metabolic syndrome and obesity are associated with increased OS, which can lead to genomic instability such as increased levels of oxidative DNA damage^24,25^. Metabolic syndrome is a collection of conditions such as deficient glucose tolerance, fatty liver, and increased body weight, adiposity, and triglyceride levels^25^. Thus, metabolic health risk could account for the observed significant difference in levels of mitochondrial 8oxoG count. Furthermore, base excision repair (BER) is a predominant DNA repair pathway for oxidative DNA damage; failure of this system allows features of genomic instability to persist and accumulate^22^. Higher 8oxoG levels in MAs may be influenced by differences in DNA repair machinery expression due to the population’s associated metabolic burden and/or population-specific variants that impact DNA repair efficiency.

Interestingly, recent evidence suggests that DNA damage repair is necessary for metabolic health, derived from observations demonstrating mtDNA repair glycosylase OGG1, an essential enzyme for BER, may influence metabolic phenotypes in high-fat diet exposure^24–26^. Functional OGG1 prevents obesity and metabolic dysfunction^24,25^ through altered *PGC-1α* expression and fatty acid oxidation^25^; reduced levels of PGC-1α has been reproducibly observed in T2D patients^2,27,28^ and have been related to increased levels of ROS and decreased levels of β-oxidation enzymes^29^. The metabolic burden in MAs may be associated with metabolic dysfunction that could alter OGG1 function causing elevated levels of 8oxoG. Interestingly, the genetic polymorphism in *OGG1* (Ser[326]Cys) has been associated with T2D risk in MAs^26^ further suggesting that insufficient response to oxidative DNA damage may be implicated in metabolic disease in the MA population.

There were significantly higher 8oxoG counts for MA females compared to MA males. In the literature, there is no clear consensus whether levels of DNA damage differ significantly based on biological sex, and this may be due to different sample types, techniques, and/or applied methods of detection across studies^30,31^. In 2014, results of a meta-analysis indicated that there are no differences between sex and DNA damage^30^. Conversely, a recent review determined that men have higher levels when compared to women; however, inconsistency in reports indicate that other factors such as lifestyle may contribute to the sex effect on the prevalence of such lesions^31^. Furthermore, most of the studies to date have not explicitly compared oxidative damage among different racial/ethnic groups in an aging population. Elevated levels of 8oxoG variant count in MA females may be partially explained by the fact that MA women have a higher frequency of T2D^32^. OS and mitochondrial dysfunction are well documented in T2D pathophysiology, and a restrictive diet reduces OS^2^. In addition, there is accumulating evidence underlining sex differences in mitochondrial function and activity, and levels of OS in an age-dependent manner^33–35^. Silaidos et al. observed that PBMCs from females exhibited significantly higher ATP levels, citrate synthase activity, uncoupled respiration, and ETC complex and system capacity when compared to PBMCs of men^33^. Recent evidence shows sex hormone status may be involved^36^. For example, mitochondrial function in female mice revealed that younger female mice display lower OS levels compared to males and that subsequent ovariectomy limited the apparent protection against DNA damage; this protection was eliminated in aged female mice^35^. The lack of consistent data in the literature regarding sex differences in aging and age-related diseases emphasizes the need for further work to better understand sex-associated disease risk, especially in ethnic populations that are rapidly expanding.

Our results from multiple linear regression analyses are suggestive of an AD-effect on 8oxoG variant count in the MA population. In the literature, there is accumulating evidence supporting the implication of mitochondrial dysfunction as a primary and/or secondary factor contributing to AD partially because of the significant levels of oxidative damage observed in various organs and tissues of individuals with cognitive impairment^4,8^. In particular, previous studies report significantly higher levels of 8oxoG and/or DNA damage in patients diagnosed with MCI or AD compared to controls, suggesting that (1) OS and subsequent DNA damage are features of AD pathophysiology, (2) accumulating oxidative DNA damage may be an early marker of AD, and (3) 8oxoG could potentially serve as a biomarker for MCI and/or AD^22,37–40^. However, there is little information regarding ethnic/racial differences in levels of oxidative DNA damage, and particularly peripheral levels of 8oxoG in the context of cognitive decline. Here we demonstrate population-specific variation in peripheral levels of mitochondrial oxidative DNA damage—the associations observed in the MA cohort were non-significant in the NHW cohort and had effect sizes in opposite directions; these findings emphasize the importance of future replication studies. As previously mentioned, it is possible that the MA population has a more pronounced effect due to their metabolic burden and potential genetic variation in DNA repair machinery. In addition, cognitive impairment has been well documented in T2D, which increases the risk for AD by two-fold and has been associated with the progression of more severe forms of cognitive impairment^7,12–14,29^. Moreover, OS is particularly related to amyloid and tau pathology through stimulating a vicious cycle of pathophysiology provoking mitochondrial dysfunction and metal toxicity, which would ultimately result in an increased mutational load and neurotoxic environment contributing to neuronal loss^8,22,40^. This gathering evidence may explain to an extent the observed suggestive association between 8oxoG variant count and AD in the MA population. Correspondingly, the stronger association reported in MA females could be attributed to the extended lifespan of women and age-related decline in sex hormones diminishing the protective effects on antioxidant defenses and mitochondrial capacity^34,41–43^. Mitochondria are responsible for steroidogenesis and its interaction with sex steroids plays an important role in the brain^42^. Brain levels of sex hormones are known to decline with age, therefore, emphasizing lifestyle factors, metabolic, health, and age may be of particular importance in accounting for the vulnerability of MA females to cognitive decline and associated pathophysiology^42^.

Interestingly, the positive association of 8oxoG variant count in MAs extended to years of education. Fletcher et al. reported associations between educational attainment and cognition in older age, after controlling for family background and genetic factors, and an interaction demonstrating those with an increased risk for AD mildly benefit from a higher educational background^44^. Educational attainment has been moderately studied in MAs with evidence indicating the disparity in cognitive impairment and dementia is due to genetic, behavioral, and socioeconomic factors^45^. Socioeconomic factors were found to be especially important in the disparity, highlighting the inequity in educational attainment among underrepresented or immigrant populations that may contribute to their risk for cognitive decline^46^. In addition, there are several reports indicating the protective effect of education on cognitive impairment does not entirely translate to MAs^47^. Data from a previous study suggests MAs may only benefit from cognitive-protective effects when years of education exceeds 12 years (i.e., education beyond high school)^47^. The reason for the observed positive association of 8oxoG with years of education in MAs is unclear; further studies investigating the effect of educational attainment on cognitive function and the paradoxical increase in 8oxoG in the MA population are warranted.

In the whole cohort, we observed mitochondrial haplogroups A and C had significantly higher 8oxoG variant counts, while haplogroups K and I showed significantly reduced levels each independently compared to all other haplogroups. Previous data have shown haplogroup K to demonstrate a protective effect against AD in European populations^48^. The significantly lower levels of 8oxoG variant count in haplogroup K may be related to its apparent low risk for developing AD that is associated with increased oxidative damage. In the NHW population, haplogroup H was found to have significantly higher levels of 8oxoG variant counts compared to all other haplogroups observed in the population. Established features of European ancestry include mtDNA haplogroups associated with largest oxygen consumption, ineffective oxygen utilization, and slightly deficient DNA repair capacity causing elevated levels of ROS that could subsequently cause elevated levels of oxidative DNA damage^49^. Furthermore, a study recently demonstrated synergism between APOE ε4 carrier status and mitochondrial haplogroup H—when combined, individuals were at higher risk for AD^50^. Therefore, the elevated 8oxoG variant count exhibited by haplogroup H that we see here was not surprising, due to their associated altered mitochondrial capacity. Conversely, in the MA population haplogroup H did not demonstrate a significant elevation in 8oxoG variant count; however, as previously mentioned the APOE risk allele appears to have less of an effect in the MA population. This observation further suggests that MAs are differentially affected by established risk factors for cognitive impairment compared to their NHW counterparts. Nonetheless, studies investigating mitochondrial haplogroup risk in neurodegeneration is very limited, and thus, it is difficult to comment on whether there is evidence to confirm or refute our findings suggesting haplogroup-specific 8oxoG variant count differences (refer to review by Ienco et al. for a comprehensive assessment of the literature)^51^. Furthermore, due to the limited sample size (i.e., various haplotypes are observed less in one population compared to the other) our power to detect rare mitochondrial haplotype effects is limited, and thus presumably causing the lack of overlap between the mitochondrial haplogroups associated with 8oxoG variant count in both cohorts. Through the historic geographical migration of certain groups and maternal nature of mtDNA inheritance, there are observed variations in haplotype frequencies between societal-based ethnic/racial groups^52^. There is accumulating evidence indicating mitonuclear allelic interactions considerably alter the expression of important health-related phenotypes by influencing the quality of oxidative phosphorylation and metabolic function^52^. Gene flow of the mitochondrial genome differs from that of the nuclear genome and considering the generation of differing genetic variation throughout populations, it is hypothesized that the course of mitonuclear coadaptation may be population specific^52^. This is likely relevant to the MA population as they are considered an admixed population. Therefore, there will be limited overlap and significant results when comparing the two populations separately, especially in relation to the whole cohort.

While our results are potentially insightful, there are several limitations to note. First, it is important to acknowledge that the methods employed here are indirect measures/indicators of oxidative damage; however, this limitation is difficult to overcome since methods for detecting oxidative damage at the per-base resolution specific to mtDNA generally have (1) technical artifacts arise during library preparation, (2) low sequencing resolution, (3) higher detection limits, and/or (4) the requirement for specific and sensitive enzymes, proteins, or antibodies^53^. Another obvious limitation is the lack of data regarding metabolic disease in this cohort; our study was limited to self-described diabetes, which is likely an oversimplification given the highly heterogenous nature of metabolic syndrome in the MA population. Furthermore, the inclusion of additional markers of metabolic health could have potentially helped with establishing an association. In general, it is challenging to interpret these results from a biological/mechanistic perspective, but importantly, they open the door for avenues of research that may prove highly relevant to addressing and resolving MA health disparities in age-related disease, namely, risk for AD.

Future studies will aim to increase the sample size and improve subject characterization of metabolic phenotypes to better resolve causal aspects of oxidative damage in MAs, specifically with respect to female vulnerability. We acknowledge that our data are suggestive in association to AD; however, future studies utilizing quantitative cognitive measures such as MMSE, CDR sum, and other measures of neuropsychological testing and cognitive function, may improve our power to support the implication. Furthermore, additional biochemical and genetic studies would solidify these results and aid in drawing conclusions. Such studies may include correlation analyses between 8oxoG variant load and expression of DNA repair machinery and ROS response systems, as genetic variant analysis of nuclear-encoded DNA repair genes and mitonuclear epistatic effects. Ideally, the studies conducted and proposed here would be recapitulated in matched blood and brain tissue to validate the potential application of these peripheral phenotypes as biomarkers for brain pathology. In addition, future studies will aim to include another population cohort and validate mitochondrial oxidative load using an alternative method such as liquid chromatography-tandem mass spectrometry.

To conclude, the work we present here describes a differential effect of oxidative mitochondrial damage that is associated with cognitive decline among MA females. We also describe a unique approach for sensitive quantification of putative oxidative damage in blood, a highly accessible tissue, and its potential relevance to cognitive aging in MAs. Furthermore, we identify a potential role for mtDNA-based haplogroup risk in 8oxoG accumulation. The systemic elevation of 8oxoG load specifically in MA females may point to an underlying source of risk for cognitive decline in this vulnerable group, revealing avenues for more precise prevention, diagnosis, and treatment of cognitive dysfunction.

## Methods

### Cohort design and samples

#### Cohort

TARCC is the Texas Alzheimer’s Research and Care Consortium, a longitudinal collaborative research initiative between ten Texas medical research institutions. The goal of TARCC is to investigate factors involved in the development and progression of AD in the MA population compared to NHWs.

#### Participants

This study was approved under the University of North Texas Health Science Center IRB #1330309-1; informed written consent was obtained from participants (or their legally authorized proxies) to take part in the study and allowing the publication of findings before data collection. Aging subjects enrolled in TARCC (*N* = 559; Table 1) who were diagnosed with AD (*n* = 104), MCI (*n* = 127), or normal cognition (*n* = 328) were selected to optimize matching with respect to age, sex and T2D distribution across MA and NHW fractions. An annual standardized assessment was conducted for each participant at one of the five original participating sites that included a medical evaluation, neuropsychological testing, an interview, and a blood draw. Buffy coat samples from 261 NHWs and 299 MAs with the aforementioned cognitive phenotypes were analyzed in this work.

### Measurement of mtDNA mutational load indicative of oxidative damage from buffy coat

#### DNA extraction

DNA was extracted from 200 μL of buffy coat sample using the Mag-Bind^®^ Blood & Tissue DNA HDQ 96 kit (Omega Bio-tek, Norcross, GA) using the Hamilton Microlab STARlet automated liquid handler (Hamilton Company, Reno, NV).

#### Whole mtDNA amplification

Whole mitochondrial genome for each sample was amplified using REPLI-g^®^ Human Mitochondrial DNA kit (Qiagen, Venlo, Netherlands) following the manufacturer’s protocol. This amplification approach follows a phi29 polymerase-based rolling circle and multiple displacement amplification. The purpose of mitochondrial genome amplification was to increase the amount of mtDNA relative to nuclear DNA to help with providing enough mtDNA for adequate coverage for whole-genome sequencing.

#### mtDNA sequencing

The Nextera XT^TM^ DNA Library Preparation kit (Illumina, San Diego, CA) was used to prepare the library for sequencing following the manufacturer’s protocol. The samples were sequenced on the NextSeq 550 Sequencer (Illumina) platform generating paired-end reads of 200 bp with an average read depth of 1855X.

#### Sequence mapping/alignment and variant calling

Raw mtDNA reads were aligned to the reference genome hg38 via BWA-MEM (v0.7.17) using the default parameter for mapping^54^. Generated SAM files were processed post-alignment with SAMtools (v.1.9) to produce BAM files that were sorted, indexed, and statistically assessed by coordinate^55^. All reads in the processed post-alignment BAM files were assigned to a single new read-group through the Picard tool AddOrReplaceReadGroups (http://broadinstitute.github.io/picard). Through GATK4 the Spark application of the Picard tool MarkDuplicates was employed on the single read-group BAM files to remove duplicate reads that may have resulted from sample preparation or the sequencing instrument^56^. BAM files with duplicate reads removed were indexed with SAMtools (v.1.9)^55^. BAM files from the previous step were used for calling somatic mutations with low allelic fractions for each sample excluding read orientation base qualities below 30 via a GATK4 tool variant caller named Mutect2 utilizing their mitochondria mode that automatically sets parameters for high-depth mitochondrial variant calling^56,57^.

#### Oxidation artifact assessment

Oxidative somatic mutations have a low allelic fraction due to their prevalence which can also be affected by tissue heterogeneity (among other factors). CollectOxoGMetrics from Picard was utilized (http://broadinstitute.github.io/picard), a tool that calculates Phred-scaled probability scores based on low allelic frequency, sequence base context, and read orientation to distinguish alternative basecalls likely resulting from a true variant from those that may result from technical oxidative damage, specifically 8oxoG (Supplementary Fig. 1). Mutational oxidative damage results from 8oxoG base-pairing with cytosine or adenine during library preparation leading to G>T or C>A transversions during PCR amplification (https://support.illumina.com). See Costello et al. for a comprehensive analysis of next-generation sequencing 8oxoG artifact generation and detection^58^. The text file outputs from each file were subjected to manual review to exclude technical oxidative artifacts. Prior to the identification of total 8oxoG variant count, all detected somatic variants for each subject were assessed for any technical oxidative variants that may have been incorrectly identified as a true variant.

#### Identification of variants indicative of oxidative damage

From the variant call files (vcf), we aimed to identify the specific mutational events that would result from oxidative damage to the template DNA mtDNA. Samples vcf files were converted to tab delimited text files through vcflib, a library collection of tools to manipulate and describe sequence variation^59^. The variant call data were imported into Excel for manual data processing in order to remove indels, transitions, and non-oxidative transversions for the selection of oxidative variants. Oxidative variants were selected based on the mutagenic property of 8oxoG mispairing with adenine ultimately resulting in the signature oxidative transversion mutations (i.e., a G, T, C, or A alternative allele call where the reference allele call was a T, G, A, or C, respectively) shown in Supplementary Fig. 2. Remaining variants indicative of oxidative damage were then further processed by removing variant calls with a read depth of less than 250 reads, removing individual SNPs (variants called in >90% reads), and removing variants where calls were limited to one orientation (forward or reverse; i.e., requiring coverage from both strands). Variants indicative of oxidative damage were summed for each sample and normalized for read depth (variant count per 1000 read depth) in both populations to test for group differences: cognitive function, sex, T2D, and comorbidity (T2D and cognitive impairment). Oxidative “hotspots” were identified as 8oxoG variant locations that occurred in at least 25 participants in the cohort.

#### Haplogroup assessment

In order to assess if background mitochondrial variants may be implicated in 8oxoG variant count, we used the NGS sequence data to derive haplogroups for statistical testing of group differences. Variant data were imported into Excel for manual processing in order to generate a list of individual SNPs for each sample (variants called in >90% reads). Each individual profile of mtDNA variants was imported into HaploGrep 2 (v.2.4.0), an online haplogroup classification tool^60^. Haplogroups were defined in our statistical analyses based on the individual’s identified macrohaplogroup or submacrohaplogroup. The sample size for this analysis was *n* = 560; one additional individual of unknown cognitive phenotype (specified as “other” and omitted from previously described analyses) was included here since this analysis is independent of cognitive phenotype.

#### Statistical analyses

Statistical analyses were performed using Microsoft Excel, IBM SPSS software (v.24.0), and R software (v. 4.0.3). Welch’s *t*-test (two-tailed) and two-way ANOVA were performed on 8oxoG mutational load to compare between both population groups and haplogroups. Multiple linear regression analysis was performed to evaluate the relationship between cognition, sex, age, education, and diabetes with 8oxoG variant count both within the whole study cohort and in stratified analyses of MAs and NHWs.

## Supplementary information

Final Supplementary material

## Data Availability

The data that support the findings of this study are subject to restrictions that limit their public availability; permission was obtained for use of these data under the current study. Data are available from the authors upon reasonable request and with permission of the Texas Alzheimer’s Research and Care Consortium’s Data Coordinating Center.

## References

[1] Alzheimer’s Association. (2021). 2021 Alzherimer’s disease facts and figures. Alzheimers Dement..

[2] Wallace DC (2005). A mitochondrial paradigm of metabolic and degenerative diseases, aging, and cancer: a dawn for evolutionary medicine. Annu. Rev. Genet..

[3] Giri M, Zhang M, Lü Y (2016). Genes associated with Alzheimer’s disease: an overview and current status. Clin. Interv. Aging.

[4] Wang X (2014). Oxidative stress and mitochondrial dysfunction in Alzheimer’s disease. Biochim. Biophys. Acta – Mol. Basis Dis..

[5] Albert MS (2011). The diagnosis of mild cognitive impairment due to Alzheimer’s disease: recommendations from the National Institute on Aging-Alzheimer’s Association workgroups on diagnostic guidelines for Alzheimer’s disease. Alzheimers Dement..

[6] McKhann GM (2011). The diagnosis of dementia due to Alzheimer’s disease: recommendations from the National Institute on Aging-Alzheimer’s Association workgroups on diagnostic guidelines for Alzheimer’s disease. Alzheimers Dement..

[7] Moreira PI, Santos MS, Seiça R, Oliveira CR (2007). Brain mitochondrial dysfunction as a link between Alzheimer’s disease and diabetes. J. Neurol. Sci..

[8] Chen Z, Zhong C (2014). Oxidative stress in Alzheimer’s disease. Neurosci. Bull..

[9] Liguori I (2018). Oxidative stress, aging, and diseases. Clin. Interv. Aging.

[10] Wallace DC (2013). A mitochondrial bioenergetic etiology of disease. J. Clin. Investig..

[11] Silzer, T. et al. Circulating mitochondrial DNA: new indices of type 2 diabetes-related cognitive impairment in Mexican Americans. *PLoS One* **14**, e0213527 (2019). 10.1371/journal.pone.0213527 PMC6414026 30861027

[12] Rad SK (2018). Mechanism involved in insulin resistance via accumulation of β-amyloid and neurofibrillary tangles: Link between type 2 diabetes and alzheimer’s disease. Drug Des. Dev. Ther..

[13] Mullins, R. J., Diehl, T. C., Chia, C. W. & Kapogiannis, D. Insulin resistance as a link between amyloid-beta and tau pathologies in Alzheimer’s disease. *Front. Aging Neurosci.* **9**, 118 (2017). 10.3389/fnagi.2017.00118 PMC5413582 28515688

[14] Gonçalves, R. A., Wijesekara, N., Fraser, P. E. & De Felice, F. G. The link between tau and insulin signaling: implications for Alzheimer’s disease and other tauopathies. *Front. Cell. Neurosci.* **13**, 17 (2019). 10.3389/fncel.2019.00017 PMC6371747 30804755

[15] Babulal GM (2019). Perspectives on ethnic and racial disparities in Alzheimer’s disease and related dementias: update and areas of immediate need. Alzheimers Dement..

[16] Johnson LA, Large SE, Izurieta Munoz H, Hall JR, O’Bryant SE (2019). Vascular depression and cognition in Mexican Americans. Dement. Geriatr. Cogn. Disord..

[17] Bertoni B, Budowle B, Sans M, Barton SA, Chakraborty R (2003). Admixture in Hispanics: distribution of ancestral population contributions in the continental United States. Hum. Biol..

[18] O’Bryant SE (2013). Biomarkers of Alzheimer’s disease among Mexican Americans. J. Alzheimers Dis..

[19] Rose KM (2005). Mild cognitive impairment in Hispanic Americans: an overview of the state of the science. Arch. Psychiatr. Nurs..

[20] Aguilar-Navarro SG (2021). Association between apoe ε4 carrier status and cardiovascular risk factors on mild cognitive impairment among mexican older adults. Brain Sci..

[21] Johnson LA (2017). Depression, inflammation, and memory loss among Mexican Americans: analysis of the HABLE cohort. Int. Psychogeriatr..

[22] Kwiatkowski D (2016). Associations between DNA damage, DNA base excision repair gene variability and Alzheimer’s disease risk. Dement. Geriatr. Cogn. Disord..

[23] Cooke MS, Olinski R, Evans MD (2006). Does measurement of oxidative damage to DNA have clinical significance?. Clinica Chimica Acta.

[24] Komakula, S. S. B. et al. The DNA repair protein OGG1 protects against obesity by altering mitochondrial energetics in white adipose tissue. *Sci. Rep.* **8**, 14886 (2018). 10.1038/s41598-018-33151-1 PMC6173743 30291284

[25] Sampath, H. et al. 8-Oxoguanine DNA Glycosylase (OGG1) deficiency increases susceptibility to obesity and metabolic dysfunction. *PLoS One* **7**, e51697 (2012). 10.1371/journal.pone.0051697 PMC3524114 23284747

[26] Thameem F (2010). The Ser(326)cys polymorphism of 8-oxoguanine glycosylase 1 (ogg1) is associated with type 2 diabetes in mexican Americans. Hum. Hered..

[27] Mootha VK (2003). PGC-1α-responsive genes involved in oxidative phosphorylation are coordinately downregulated in human diabetes. Nat. Genet..

[28] Patti ME (2003). Coordinated reduction of genes of oxidative metabolism in humans with insulin resistance and diabetes: potential role of PGC1 and NRF1. Proc. Natl. Acad. Sci. U.S.A..

[29] Zilliox, L. A., Chadrasekaran, K., Kwan, J. Y. & Russell, J. W. Diabetes and cognitive impairment. *Curr. Diab. Rep.* **16**, 87 (2016). 10.1007/s11892-016-0775-x PMC5528145 27491830

[30] Soares JP (2014). Aging and DNA damage in humans: a meta-analysis study. Aging.

[31] Møller P (2019). Effect of age and sex on the level of DNA strand breaks and oxidatively damaged DNA in human blood cells. Mut. Res. – Genet. Toxicol. Environ. Mutagen..

[32] Martorell, R. Diabetes and Mexicans: why the two are linked. *Prev. Chronic Dis.* **2**, A04 (2005). PMC1323307 15670457

[33] Silaidos, C. et al. Sex-associated differences in mitochondrial function in human peripheral blood mononuclear cells (PBMCs) and brain. *Biol. Sex Differ.* **9**, 34 (2018). 10.1186/s13293-018-0193-7 PMC6060503 30045765

[34] Ventura-Clapier R (2017). Mitochondria: a central target for sex differences in pathologies. Clin. Sci..

[35] Gaignard P (2015). Effect of sex differences on brain mitochondrial function and its suppression by ovariectomy and in aged mice. Endocrinology.

[36] Duong, P. et al. Neuroprotective and neurotoxic outcomes of androgens and estrogens in an oxidative stress environment. *Biol. Sex Differ.* **11**, 12 (2020). 10.1186/s13293-020-0283-1 PMC7104511 32223745

[37] Migliore L (2005). Oxidative DNA damage in peripheral leukocytes of mild cognitive impairment and AD patients. Neurobiol. Aging.

[38] Wang J, Markesbery WR, Lovell MA (2006). Increased oxidative damage in nuclear and mitochondrial DNA in mild cognitive impairment. J. Neurochem..

[39] Torres LL (2011). Peripheral oxidative stress biomarkers in mild cognitive impairment and alzheimer’s disease. J. Alzheimers Dis..

[40] Sliwinska A (2016). The levels of 7,8-dihydrodeoxyguanosine (8-oxoG) and 8-oxoguanine DNA glycosylase 1 (OGG1) – a potential diagnostic biomarkers of Alzheimer’s disease. J. Neurol. Sci..

[41] Andrew, M. K. & Tierney, M. C. The puzzle of sex, gender and Alzheimer’s disease: why are women more often affected than men? *Womens Health* **14**, 1745506518817995 (2018).

[42] Gaignard, P. et al. Role of sex hormones on brain mitochondrial function, with special reference to aging and neurodegenerative diseases. *Front. Aging Neurosci.* **9**, 406 (2017). 10.3389/fnagi.2017.00406 PMC5725410 29270123

[43] Garcia MA (2018). Age of migration differentials in life expectancy with cognitive impairment: 20-year findings from the Hispanic-EPESE. Gerontologist.

[44] Fletcher, J., Topping, M., Zheng, F. & Lu, Q. The effects of education on cognition in older age: evidence from genotyped Siblings. *Soc. Sci. Med.* **280**, 114044 (2021). 10.1016/j.socscimed.2021.114044 PMC8205990 34029863

[45] Rote SM, Angel JL (2021). Gender-based pathways to cognitive aging in the Mexican-origin population in the United States: the significance of work and family. J. Gerontol. B Psychol. Sci. Soc. Sci..

[46] Garcia MA, Saenz J, Downer B, Wong R (2018). The role of education in the association between race/ethnicity/nativity, cognitive impairment, and dementia among older adults in the United States. Demographic Res..

[47] O’Bryant SE (2013). Risk factors for mild cognitive impairment among Mexican Americans. Alzheimers Dement..

[48] Carrieri G (2001). Mitochondrial DNA haplogroups and APOE4 allele are non-independent variables in sporadic Alzheimer’s disease. Hum. Genet..

[49] Nayyar, A. & Chakalova, L. APOE4, oxidative stress and decreased repair capacity – a no-brainer. Faulty lipid metabolism and increased levels of oxidative damage may be risk factors in the pathogenesis of late-onset dementia. *BioDiscovery* **17**, e8969 10.7750/biodiscovery.2015.17.1 (2015).

[50] Wang, Y. & Brinton, R. D. Triad of risk for late onset Alzheimer’s: mitochondrial haplotype, apoe genotype and chromosomal sex. *Front. Aging Neurosci*. **8**, 232 (2016). 10.3389/fnagi.2016.00232 PMC5047907 27757081

[51] Mancuso, M. et al. May mitochondrial eve and mitochondrial haplogroups play a role in neurodegeneration and alzheimer’s disease? *Int. J. Alzheimers Dis.* **2011**, 709061. 10.4061/2011/709061 (2011). 10.4061/2011/709061 PMC3056451 21423558

[52] Wolff, J. N., Ladoukakis, E. D., Enríquez, J. A. & Dowling, D. K. Mitonuclear interactions: evolutionary consequences over multiple biological scales. *Philos. Trans. R Soc. Lond. B Biol. Sci.* **369**, 20130443 (2014). 10.1098/rstb.2013.0443 PMC4032519 24864313

[53] Li W, Sancar A (2020). Methodologies for detecting environmentally induced DNA damage and repair. Environ. Mol. Mutagen..

[54] Li, H. Aligning sequence reads, clone sequences and assembly contigs with BWA-MEM. Preprint at *arxiv* http://arxiv.org/abs/1303.3997 (2013).

[55] Danecek, P. et al. Twelve years of SAMtools and BCFtools. *GigaScience* **10**, giab008 (2021). 10.1093/gigascience/giab008 PMC7931819 33590861

[56] Van der Auwera, G. A. & O’Connor, B. *Genomics in the Cloud: Using Docker, GATK, and WDL in Terra* (O’Reilly Media, Inc., 2020).

[57] Benjamin, D. et al. Calling somatic SNVs and indels with Mutect2. Preprint at *bioRxiv*10.1101/861054 (2019).

[58] Costello M (2013). Discovery and characterization of artifactual mutations in deep coverage targeted capture sequencing data due to oxidative DNA damage during sample preparation. Nucleic Acids Res..

[59] Garrison, E., Kronenberg, Z. N., Dawson, E. T., Pedersen, B. S. & Prins, P. Vcflib and tools for processing the VCF variant call format. Preprint at *bioRxiv* 2021.05.21.445151 (2021). 10.1371/journal.pcbi.1009123 PMC9286226 35639788

[60] Weissensteiner H (2016). HaploGrep 2: mitochondrial haplogroup classification in the era of high-throughput sequencing. Nucleic Acids Res..

[61] van Oven M, Kayser M (2009). Updated comprehensive phylogenetic tree of global human mitochondrial DNA variation. Hum. Mutat..

