# Supplementary material for: Mitochondrial DNA oxidative mutations are elevated in Mexican American women potentially implicating Alzheimer’s disease
Source: NPJ Aging. 2022 Apr 4;8(1):2. doi: 10.1038/s41514-022-00082-1 (PMC9158774; doi:10.1038/s41514-022-00082-1)
Supplement: Supplementary file 1 — Final Supplementary material [file 41514_2022_82_MOESM1_ESM.pdf]

Supplemental Materials

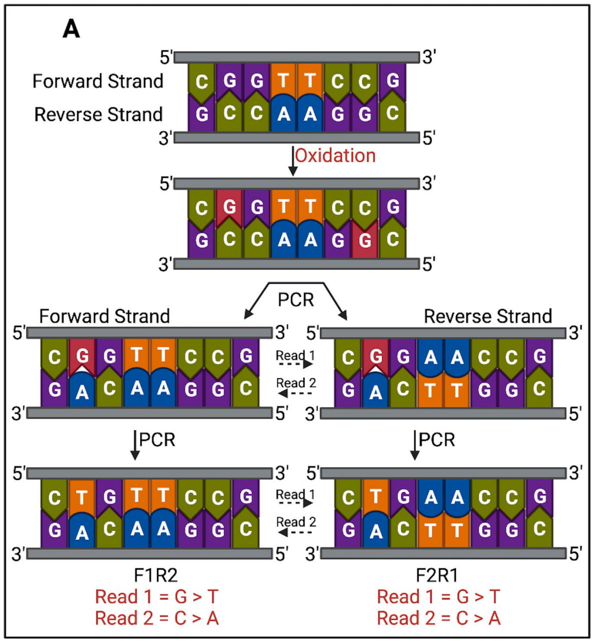

Supplementary Figure 1. Next-generation sequencing read strand orientation bias and resulting artifacts. The presence of artifactual C>A/G>T transversions in sequencing data arises from the oxidation of guanine prior to PCR amplification. During PCR amplification adenine is misincorporated base-pairing with 8oxoG. Subsequent amplification steps produce the transversion mutations. Due to the nature of Illumina NGS chemistry, the G>T transversion will sequence on read 1 and the C>A transversion will sequence on read 2. This figure was created with BioRender.com.

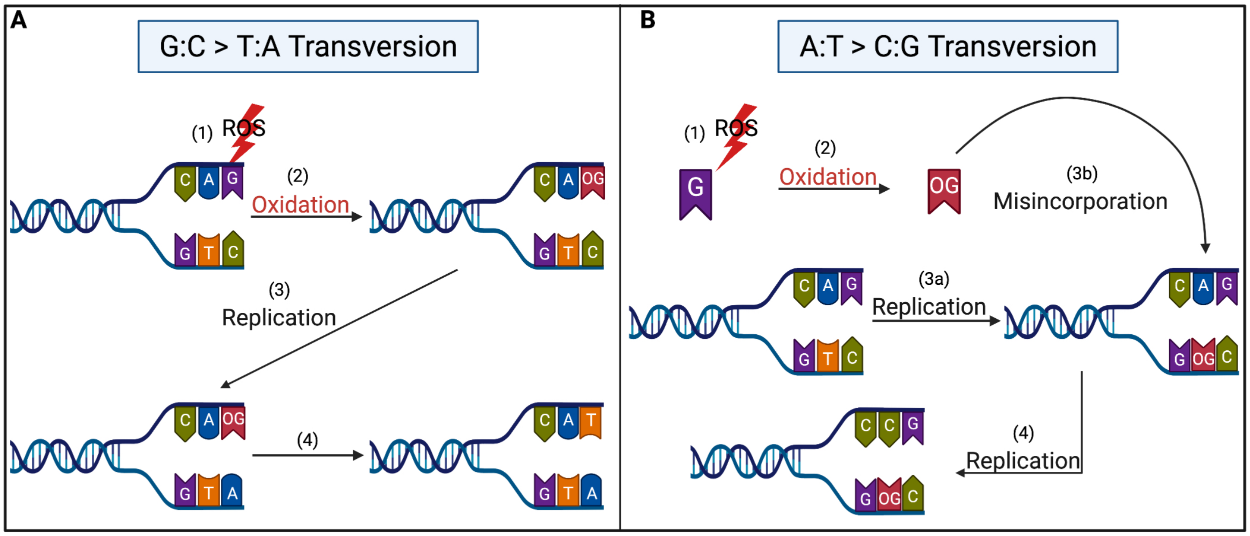

Supplementary Figure 2. Overview of oxidized guanine mutagenesis in DNA. Oxidized guanine (8-oxoG or 8-oxodG) has mutagenic capabilities whether it is in native DNA or a free-floating nucleotide in the deoxynucleotide pool to be incorporated during replication. A. Oxidation of G in native double strand DNA (dsDNA) causes the modified base to be paired opposite deoxycytidine. Subsequent replication without the removal of 8-oxoG in the template strand by BER or other DNA repair pathways can result in the mispairing of deoxyadenosine opposite the modified base. Further replication of the dsDNA may result in deoxythymidine to be correctly paired with deoxyadenosine causing a G:C to T:A transversion. B. Oxidation of guanine in the deoxynucleotide pool can be misincorporated opposite deoxyadenosine during replication. An additional round of replication can incorporate deoxycytidine opposite the oxidized base resulting in a A:T to C:G transversion. This figure was created with BioRender.com.

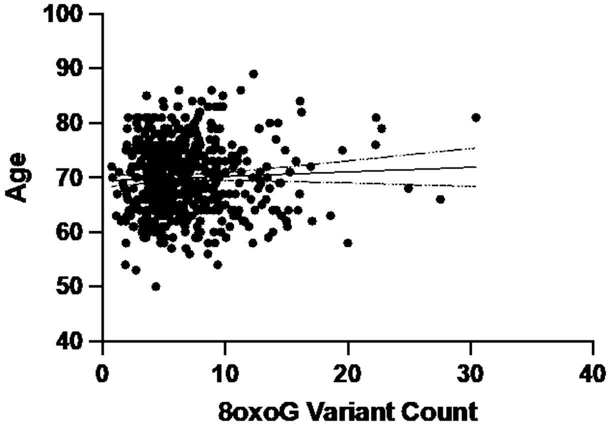

Supplementary Figure 3. Scatter plot of 8oxoG variant count by age. Sample means of total 8oxoG variant count was assessed by age using a two-tailed Pearson correlation (n = 559). Dotted lines represent 95% confidence interval (-0.03757 to 0.1333), and the solid line indicates best-fit line. Correlation statistics: r = 0.04824, R squared = 0.002327, p-value = 0.2704.

Supplementary Table 1. 8oxoG variant count multiple linear regression model prediction considering cognitive status (NC vs MCI or AD) and diabetes interaction effect. Italics and bolding indicate a *p*-value of significance, while italics alone indicate a *p*-value approaching significance.

Variable	Coefficient	Std. Error	t-statistic	p-value
Constant	2.065866	2.198627	0.94	0.34783
Population (with respect to NHW)	-1.959649	0.406268	-4.824	***1.84E-06***
Cognitive Status (with respect to AD)	1.086334	0.570781	1.903	*0.05754*
Cognitive Status (with respect to MCI)	0.50188	0.525202	0.956	0.3397
Sex (with respect to Male)	-0.68253	0.322023	-2.12	***0.0345***
Diabetes (with respect to “Yes”)	-0.009736	0.444694	-0.022	0.98254
APOE ε2/ε2	-1.464225	2.70427	-0.541	0.58842
APOE ε2/ε3	0.84649	0.813543	1.04	0.29857
APOE ε2/ε4	0.138294	2.235666	0.062	0.9507
APOE ε3/ε3	0.716064	0.620893	1.153	0.2493
APOE ε3/ε4	0.380755	0.670123	0.568	0.57014
APOE ε4/ε4	0.989753	0.918605	1.077	0.28176
BMI	0.035386	0.02488	1.422	0.15552
Years of Education	0.107933	0.041059	2.629	***0.00881***
Age	0.039031	0.025168	1.551	0.12154
Interaction: AD x Diabetes “Yes”	-1.170123	0.864129	-1.354	0.17627
Interaction: MCI x Diabetes “Yes”	-0.224683	0.807738	-0.278	0.78099
R-squared	0.07337		*p*-value	4.185e-04
Adjusted R-squared	0.04601		df	16 and 542
F-statistic	2.682		Sample n	559

Supplementary Table 2. 8oxoG variant count multiple linear regression model prediction considering cognitive impairment (NC vs MCI + AD) and diabetes interaction effect. Italics and bolding indicate a *p*-value of significance, while italics alone indicate a *p*-value approaching significance.

Variable	Coefficient	Std. Error	t-statistic	p-value
Constant	2.058131	2.195539	0.937	0.34896
Population (with respect to NHW)	-1.927984	0.404489	-4.766	***2.41E-06***
Cognitive Impairment	0.76542	0.439084	1.743	*0.08186*
Sex (with respect to Male)	-0.685558	0.321389	-2.133	***0.03336***
Diabetes (with respect to "Yes")	-0.003315	0.444255	-0.007	0.99405
APOE ε2/ε2	-1.469991	2.701135	-0.544	0.58652
APOE ε2/ε3	0.805548	0.808028	0.997	0.31924
APOE ε2/ε4	0.111867	2.231898	0.05	0.96004
APOE ε3/ε3	0.71578	0.616826	1.16	0.24638
APOE ε3/ε4	0.408495	0.668919	0.611	0.54167
APOE ε4/ε4	0.95647	0.916872	1.043	0.29732
BMI	0.034368	0.024827	1.384	0.16683
Years of Education	0.109596	0.040924	2.678	***0.00763***
Age	0.039027	0.025094	1.555	0.12048
Interaction: Cognitive Impairment x Diabetes "Yes"	-0.653041	0.663536	-0.984	0.32546

R-squared	0.07159		*p*-value	1.875e-04
Adjusted R-squared	0.0477		df	14 and 544
F-statistic	2.997		Sample n	559

Supplementary Table 3. 8oxoG variant count and cognitive impairment (NC vs MCI + AD) multiple linear regression model prediction considering population interaction effect with both sex and education. Italics and bolding indicate a *p*-value of significance, while italics alone indicate a *p*-value approaching significance.

Variable	Coefficient	Std. Error	t-statistic	p-value
Constant	2.88116	2.21084	1.303	0.19306
Population (with respect to NHW)	-0.51694	1.46467	-0.353	0.72427
Cognitive Impairment	0.49811	0.34392	1.448	0.1481
Sex (with respect to Male)	-1.42915	0.44298	-3.226	***0.00133***
Diabetes (with respect to "Yes")	-0.35695	0.33806	-1.056	0.2915
Years of Education	0.1441	0.04539	3.175	***0.00158***
APOE ε2/ε2	-2.34923	2.69473	-0.872	0.38371
APOE ε2/ε3	0.62346	0.80435	0.775	0.43861
APOE ε2/ε4	0.46293	2.23149	0.207	0.83573
APOE ε3/ε3	0.6054	0.60887	0.994	0.32051
APOE ε3/ε4	0.36706	0.66375	0.553	0.58049
APOE ε4/ε4	0.65263	0.91027	0.717	0.4737
BMI	0.03332	0.02463	1.353	0.17657
Age	0.03107	0.02514	1.236	0.2171
Interaction: NHW x Male "Yes"	1.59823	0.64596	2.474	***0.01366***
Interaction: NHW x Years of Education	-0.15208	0.09796	-1.553	0.12111
R-squared	0.08267		*p*-value	3.163e-05
Adjusted R-squared	0.05733		df	15 and 543
F-statistic	3.262		Sample n	559

Supplementary Table 4. 8oxoG variant count stratification in the Mexican American population by cognitive impairment (NC vs MCI + AD). Italics and bolding indicate a *p*-value of significance, while italics alone indicate a *p*-value approaching significance.

Variable	Coefficient	Std. Error	t-statistic	p-value
Constant	5.09069	3.63725	1.4	0.16271
Cognitive Impairment	0.95919	0.53876	1.78	*0.07607*
Sex (with respect to Male)	-1.43742	0.52445	-2.741	***0.00651***
Diabetes (with respect to "Yes")	-0.24172	0.52806	-0.458	0.64747
APOE ε2/ε2	-1.85575	4.71698	-0.393	0.6943
APOE ε2/ε3	0.06738	2.16343	0.031	0.97517
APOE ε3/ε3	-0.60564	1.95648	-0.31	0.75712
APOE ε3/ε4	-0.80412	2.00258	-0.402	0.68832
APOE ε4/ε4	-1.07929	2.90559	-0.371	0.71057
BMI	0.02298	0.03896	0.59	0.55584
Years of Education	0.14618	0.05408	2.703	***0.00728***
Age	0.01642	0.03892	0.422	0.67335

R-squared	0.05647	*p*-value	0.1096
Adjusted R-squared	0.02031	df	11 and 287
F-statistic	1.562	Sample n	299

Supplementary Table 5. Stratification analysis in NHWs for 8oxoG variant count by cognitive impairment (NC vs MCI or AD). Italics and bolding indicate a *p*-value of significance, while italics alone indicate a *p*-value approaching significance.

**Variable**	**Coefficient**	**Std. Error**	**t-statistic**	**p-value**
Constant	0.61747	2.80517	0.22	0.826
Cognitive Impairment	-0.11555	0.41685	-0.277	0.7819
Sex (with respect to Male)	0.18141	0.37302	0.486	0.6272
Diabetes (with respect to "Yes")	-0.54057	0.40557	-1.333	0.1838
APOE ε2/ε2	-4.01421	2.98532	-1.345	0.18
APOE ε2/ε3	0.37297	0.79069	0.472	0.6376
APOE ε2/ε4	0.34083	1.78683	0.191	0.8489
APOE ε3/ε3	0.93935	0.5389	1.743	*0.0826*
APOE ε3/ε4	0.76551	0.65468	1.169	0.2434
APOE ε4/ε4	1.29813	0.81143	1.6	0.1109
BMI	0.04823	0.02878	1.676	*0.095*
Years of Education	-0.02983	0.07078	-0.421	0.6738
Age	0.0556	0.03095	1.796	*0.0736*

R-squared	0.04768	*p*-value	0.4212
Adjusted R-squared	0.001411	df	12 and 247
F-statistic	1.03	Sample n	260

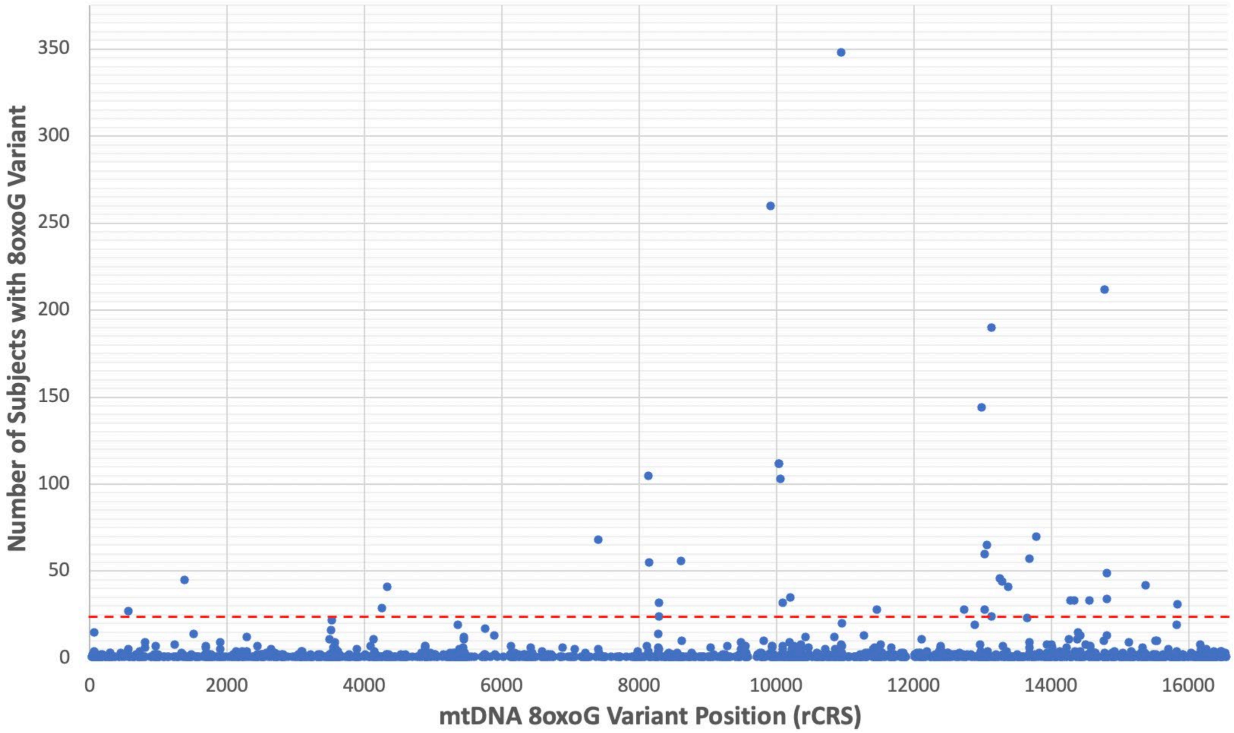

Supplementary Figure 4. Distribution of 8oxoG variant in mitochondrial genome by number of individuals with 8oxoG variant- 8oxoG "hotspot" variant selection. The red dashed line intercepts at 25 subjects with 8oxoG variant. 8oxoG variants above the intercept were conveniently selected as "hotspots".

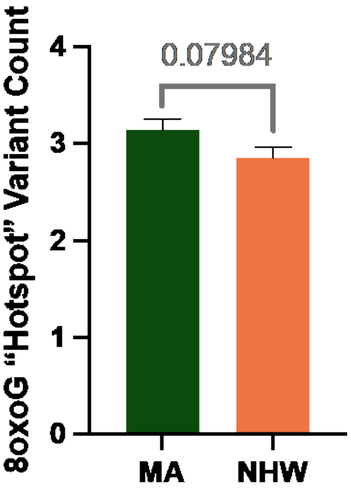

Supplementary Figure 5. 8oxoG “hotspot” variant count does not differ between populations. Welch’s t-test was performed to determine statistical difference in 8oxoG “hotspot” variant count between MAs and NHWs (n = 559). Error bars represent standard error of the mean.

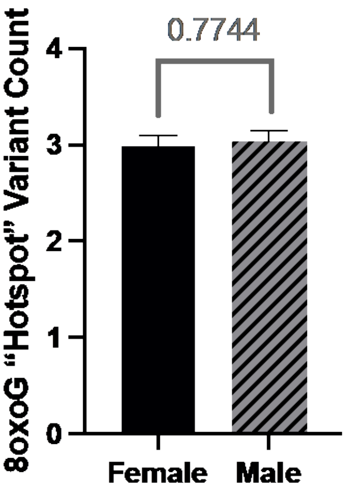

Supplementary Figure 6. 8oxoG “hotspot” variant count does not differ between sexes. Welch’s t-test was performed on 8oxoG “hotspot” variant count by sex (n =559). Error bars represent standard error of the mean.

Supplementary Table 6. 8oxoG "hotspot" variant count multiple linear regression model prediction considering cognitive status (NC vs MCI or AD) and diabetes interaction effect. Italics and bolding indicate a *p*-value of significance, while italics alone indicate a *p*-value approaching significance.

Variable	Coefficient	Std. Error	t-statistic	p-value
Constant	1.031513	1.106376	0.932	0.3516
Population with respect to NHW	-0.236656	0.204439	-1.158	0.2475
Cognitive Status with respect to AD	0.034295	0.287224	0.119	0.905
Cognitive Status with respect to MCI	0.09451	0.264288	0.358	0.7208
Sex with respect to Male	0.077441	0.162046	0.478	0.6329
Diabetes with respect to "Yes"	0.089844	0.223776	0.401	0.6882
APOE ε2/ε2	0.462665	1.360822	0.34	0.734
APOE ε2/ε3	0.662786	0.409385	1.619	0.106
APOE ε2/ε4	1.064559	1.125014	0.946	0.3444
APOE ε3/ε3	0.623034	0.312441	1.994	***0.0466***
APOE ε3/ε4	0.606919	0.337214	1.8	*0.0724*
APOE ε4/ε4	0.963228	0.462253	2.084	***0.0376***
BMI	0.016726	0.01252	1.336	0.1821
Years of Education	0.007475	0.020661	0.362	0.7177
Age	0.012951	0.012665	1.023	0.307
Interaction: AD x Diabetes "Yes"	-0.182847	0.43484	-0.42	0.6743
Interaction: MCI x Diabetes "Yes"	-0.767987	0.406463	-1.889	*0.0594*
R-squared	0.02788		*p*-value	0.4869
Adjusted R-squared	-0.0008199		df	16 and 542
F-statistic	0.9714		Sample n	559

Supplementary Table 7. 8oxoG “hotspot” variant count multiple linear regression model prediction considering cognitive impairment (NC vs MCI + AD) and diabetes interaction effect. Italics and bolding indicate a *p*-value of significance, while italics alone indicate a *p*-value approaching significance.

Variable	Coefficient	Std. Error	t-statistic	p-value
Constant	0.996468	1.105562	0.901	0.3678
Population with respect to NHW	-0.241563	0.20368	-1.186	0.2361
Cognitive Impairment	0.060094	0.221101	0.272	0.7859
Sex with respect to Male	0.070685	0.161835	0.437	0.6624
Diabetes with respect to "Yes"	0.086946	0.223705	0.389	0.6977
APOE ε2/ε2	0.430834	1.360154	0.317	0.7516
APOE ε2/ε3	0.636282	0.406882	1.564	0.1184
APOE ε2/ε4	1.028611	1.123871	0.915	0.3605
APOE ε3/ε3	0.587519	0.310602	1.892	*0.0591*
APOE ε3/ε4	0.591401	0.336834	1.756	*0.0797*
APOE ε4/ε4	0.989615	0.46169	2.143	***0.0325***
BMI	0.016723	0.012502	1.338	0.1816
Years of Education	0.008087	0.020607	0.392	0.6949
Age	0.013824	0.012636	1.094	0.2744
Interaction: Cognitive Impairment x Diabetes "Yes"	-0.501576	0.334123	-1.501	0.1339

R-squared	0.02471		*p*-value	0.4677
Adjusted R-squared	-0.0003888		df	14 and 544
F-statistic	0.9845		Sample n	559

Supplementary Table 8. 8oxoG "hotspot" variant count and cognitive status (NC vs MCI or AD) multiple linear regression model prediction considering population interaction effect with both sex and education. Italics and bolding indicate a *p*-value of significance, while italics alone indicate a *p*-value approaching significance.

Variable	Coefficient	Std. Error	t-statistic	p-value
Constant	2.246331	0.974249	2.306	***0.0215***
Population with respect to NHW	-0.753823	0.743259	-1.014	0.3109
Cognitive Status with respect to AD	-0.055	0.228654	-0.241	0.81
Cognitive Status with respect to MCI	-0.185378	0.206759	-0.897	0.3703
Sex with respect to Male	-0.163209	0.22485	-0.726	0.4682
Diabetes with respect to "Yes"	-0.089799	0.167338	-0.537	0.5917
Years of Education	0.002068	0.023077	0.09	0.9286
APOE ε2/ε2	0.223413	1.367797	0.163	0.8703
APOE ε2/ε3	0.578424	0.410699	1.408	0.1596
APOE ε2/ε4	0.949603	1.132505	0.838	0.4021
APOE ε3/ε3	0.514769	0.310391	1.658	*0.0978*
APOE ε3/ε4	0.545231	0.33675	1.619	0.106
APOE ε4/ε4	0.853611	0.461773	1.849	*0.0651*
Age	0.007646	0.012544	0.61	0.5424
Interaction: NHW x Male "Yes"	0.471257	0.328227	1.436	0.1516
Interaction: NHW x Years of Education	0.016038	0.049696	0.323	0.747
R-squared	0.02163		*p*-value	0.6776
Adjusted R-squared	-0.005392		df	15 and 543
F-statistic	0.8005		Sample n	559

Supplementary Table 9. 8oxoG "hotspot" variant count and cognitive impairment (NC vs MCI + AD) multiple linear regression model prediction considering population interaction effect with both sex and education. Italics and bolding indicate a *p*-value of significance, while italics alone indicate a *p*-value approaching significance.

Variable	Coefficient	Std. Error	t-statistic	p-value
Constant	2.221287	0.972313	2.285	***0.0227***
Population with respect to NHW	-0.743506	0.742467	-1.001	0.3171
Cognitive Impairment	-0.129473	0.174356	-0.743	0.4581
Sex with respect to Male	-0.172106	0.224002	-0.768	0.4426
Diabetes with respect to "Yes"	-0.09046	0.167219	-0.541	0.5888
Years of Education	0.002985	0.02299	0.13	0.8967
APOE ε2/ε2	0.203655	1.366297	0.149	0.8816
APOE ε2/ε3	0.555142	0.407812	1.361	0.174
APOE ε2/ε4	0.928906	1.130984	0.821	0.4118
APOE ε3/ε3	0.499258	0.30865	1.618	0.1063
APOE ε3/ε4	0.546107	0.336514	1.623	0.1052
APOE ε4/ε4	0.854969	0.461449	1.853	*0.0645*
Age	0.008026	0.012513	0.641	0.5215
Interaction: NHW x Male "Yes"	0.482702	0.327216	1.475	0.1407
Interaction: NHW x Years of Education	0.015416	0.049646	0.311	0.7563
R-squared	0.02118		*p*-value	0.6246
Adjusted R-squared	-0.004013		df	14 and 544
F-statistic	0.8407		Sample n	559

Supplementary Table 10. Multiple linear regression results for 8oxoG "hotspot" variant count and cognitive status (NC vs MCI or AD) within Mexican Americans. Italics and bolding indicate a p-value of significance, while italics alone indicate a p-value approaching significance.

Variable	Coefficient	Std. Error	t-statistic	p-value
Constant	3.119633	1.728499	1.805	*0.0722*
Cognitive Status (with respect to AD)	-0.18077	0.382078	-0.473	0.6365
Cognitive Status (with respect to MCI)	-0.427275	0.2831	-1.509	0.1323
Sex (with respect to Male)	-0.099865	0.250312	-0.399	0.6902
Diabetes (with respect to "Yes")	-0.04695	0.251607	-0.187	0.8521
APOE ε2/ε2	0.721106	2.243469	0.321	0.7481
APOE ε2/ε3	-0.64859	1.032714	-0.628	0.5305
APOE ε3/ε3	-0.711241	0.933457	-0.762	0.4467
APOE ε3/ε4	-0.583709	0.95293	-0.613	0.5407
APOE ε4/ε4	-0.060057	1.388688	-0.043	0.9655
BMI	0.003853	0.018646	0.207	0.8364
Years of Education	-0.007986	0.025716	-0.311	0.7564
Age	0.012322	0.018637	0.661	0.509
R-squared	0.01516		*p*-value	0.974
Adjusted R-squared	-0.02616		df	12 and 286
F-statistic	0.3669		Sample n	299

Supplementary Table 11. Multiple linear regression results for 8oxoG "hotspot" variant count and cognitive impairment (NC vs MCI + AD) within Mexican Americans. Italics and bolding indicate a p-value of significance, while italics alone indicate a p-value approaching significance.

Variable	Coefficient	Std. Error	t-statistic	p-value
Constant	3.108745	1.726513	1.801	*0.0728*
Cognitive Impairment	-0.353629	0.255736	-1.383	0.1678
Sex (with respect to Male)	-0.114114	0.248945	-0.458	0.647
Diabetes (with respect to "Yes")	-0.035737	0.250658	-0.143	0.8867
APOE ε2/ε2	0.663692	2.239034	0.296	0.7671
APOE ε2/ε3	-0.708339	1.026924	-0.69	0.4909
APOE ε3/ε3	-0.762167	0.928691	-0.821	0.4125
APOE ε3/ε4	-0.61417	0.950576	-0.646	0.5187
APOE ε4/ε4	-0.15056	1.379213	-0.109	0.9131
BMI	0.0025	0.018493	0.135	0.8926
Years of Education	-0.007422	0.025671	-0.289	0.7727
Age	0.013734	0.018472	0.743	0.4578

R-squared	0.01388	*p*-value	0.9677
Adjusted R-squared	-0.02392	df	11 and 287
F-statistic	0.3672	Sample n	299

Supplementary Table 12. Multiple linear regression results for 8oxoG “hotspot” variant count and cognitive status (NC vs MCI or AD) within non-Hispanic Whites. Italics and bolding indicate a p-value of significance, while italics alone indicate a p-value approaching significance.

**Variable**	**Coefficient**	**Std. Error**	**t-statistic**	**p-value**
Constant	-0.37463	1.60903	-0.233	0.8161
Cognitive Status (with respect to AD)	0.05397	0.2804	0.192	0.8475
Cognitive Status (with respect to MCI)	0.19302	0.30975	0.623	0.5338
Sex (with respect to Male)	0.30995	0.21397	1.449	0.1487
Diabetes (with respect to "Yes")	-0.31488	0.23322	-1.35	0.1782
APOE ε2/ε2	-1.33584	1.71249	-0.78	0.4361
APOE ε2/ε3	0.63187	0.46154	1.369	0.1722
APOE ε2/ε4	0.93667	1.02604	0.913	0.3622
APOE ε3/ε3	0.6952	0.31339	2.218	***0.0274***
APOE ε3/ε4	0.52071	0.37546	1.387	0.1667
APOE ε4/ε4	0.80166	0.46547	1.722	*0.0863*
BMI	0.03831	0.01651	2.321	***0.0211***
Years of Education	0.03061	0.04068	0.753	0.4524
Age	0.01545	0.01775	0.87	0.3849
R-squared	0.06899		*p*-value	0.1585
Adjusted R-squared	0.01979		df	13 and 246
F-statistic	1.402		Sample n	260

Supplementary Table 13. Multiple linear regression results for 8oxoG “hotspot” variant count and cognitive impairment (NC vs MCI + AD) within non-Hispanic Whites. Italics and bolding indicate a p-value of significance, while italics alone indicate a p-value approaching significance.

Variable	Coefficient	Std. Error	t-statistic	p-value
Constant	-0.36081	1.60594	-0.225	0.8224
Cognitive Impairment	0.11329	0.23864	0.475	0.6354
Sex (with respect to Male)	0.30798	0.21355	1.442	0.1505
Diabetes (with respect to "Yes")	-0.30786	0.23218	-1.326	0.1861
APOE ε2/ε2	-1.31871	1.70907	-0.772	0.4411
APOE ε2/ε3	0.66673	0.45266	1.473	0.1421
APOE ε2/ε4	0.95804	1.02294	0.937	0.3499
APOE ε3/ε3	0.71626	0.30852	2.322	***0.0211***
APOE ε3/ε4	0.52254	0.3748	1.394	0.1645
APOE ε4/ε4	0.80645	0.46454	1.736	*0.0838*
BMI	0.03815	0.01647	2.316	***0.0214***
Years of Education	0.02952	0.04052	0.728	0.467
Age	0.01533	0.01772	0.865	0.3877

R-squared	0.06837	*p*-value	0.1206
Adjusted R-squared	0.02311	df	12 and 247
F-statistic	1.511	Sample n	260
